# Nanostructural Diversity of Synapses in the Mammalian Spinal Cord

**DOI:** 10.1038/s41598-020-64874-9

**Published:** 2020-05-18

**Authors:** Matthew J. Broadhead, Calum Bonthron, Lauren Arcinas, Sumi Bez, Fei Zhu, Frances Goff, Jonathan Nylk, Kishan Dholakia, Frank Gunn-Moore, Seth G. N. Grant, Gareth B. Miles

**Affiliations:** 1 School of Psychology and Neuroscience, University of St Andrews, St Andrews, UK; 2 Edinburgh Super-Resolution Imaging Consortium, Heriot Watt University, Edinburgh, UK; 3 Genes to Cognition Programme, Centre for Clinical Brain Sciences, University of Edinburgh, Edinburgh, UK; 4 School of Biology, University of St Andrews, St Andrews, UK; 5 SUPA, School of Physics and Astronomy, University of St Andrews, St Andrews, UK; 6 Centre for Biophotonics, North Haugh, University of St Andrews, St Andrews, UK

**Keywords:** Astrocyte, Neural circuits, Spine regulation and structure

## Abstract

Functionally distinct synapses exhibit diverse and complex organisation at molecular and nanoscale levels. Synaptic diversity may be dependent on developmental stage, anatomical locus and the neural circuit within which synapses reside. Furthermore, astrocytes, which align with pre and post-synaptic structures to form ‘tripartite synapses’, can modulate neural circuits and impact on synaptic organisation. In this study, we aimed to determine which factors impact the diversity of excitatory synapses throughout the lumbar spinal cord. We used PSD95-eGFP mice, to visualise excitatory postsynaptic densities (PSDs) using high-resolution and super-resolution microscopy. We reveal a detailed and quantitative map of the features of excitatory synapses in the lumbar spinal cord, detailing synaptic diversity that is dependent on developmental stage, anatomical region and whether associated with VGLUT1 or VGLUT2 terminals. We report that PSDs are nanostructurally distinct between spinal laminae and across age groups. PSDs receiving VGLUT1 inputs also show enhanced nanostructural complexity compared with those receiving VGLUT2 inputs, suggesting pathway-specific diversity. Finally, we show that PSDs exhibit greater nanostructural complexity when part of tripartite synapses, and we provide evidence that astrocytic activation enhances PSD95 expression. Taken together, these results provide novel insights into the regulation and diversification of synapses across functionally distinct spinal regions and advance our general understanding of the ‘rules’ governing synaptic nanostructural organisation.

## Introduction

Neuronal synapses show considerable structural, molecular and functional diversity throughout the nervous system. Their morphology, molecular composition and nanostructural organisation determines synaptic strength and function^1,2^, and must therefore be finely tuned to the circuits within which they operate^3^. Synapse morphology may be determined by a number of factors, such as the type of neuron, the anatomical location, the activity state of the network, and development and ageing^4–9^. In order to understand the basic logic of different neural circuits, it is pertinent to study the principles of synaptic morphology that underlie the function of neural circuits. Such information may help produce a set of basic ‘rules’ governing synaptic structure and therefore function.

Key scaffolding proteins such as PSD95, one of the most abundantly expressed proteins from the Dlg family, form the core molecular architecture of the synapse^10–13^. At a molecular level, PSD95 interacts with a host of different signalling enzymes and neurotransmitter receptors within the postsynaptic density (PSD) to form PSD95-dependent super-complexes (~1.5MDa in weight)^14–17^. These super-complexes are heterogeneously distributed within the PSD, forming protein-enriched domains known as nanoclusters (NCs, ~140 nm diameter)^4,5,18,19^, which align with presynaptic release sites to form trans-synaptic nanocolumns^1^. Visualising PSD95 enables us to determine the nanostructural and molecular architecture of the vast majority of excitatory PSDs^20^.

While there is considerable data on the diversity of synapses in different regions of the brain, such as the hippocampus and cortex^4,8,20,21^, less is known about synaptic diversity in the spinal cord. Previous studies characterising and differentiating spinal synaptic inputs have largely focussed either on synapses solely onto motor neurons^22–25^ or synapses within pain and sensory processing circuitry of the dorsal horn^26–29^. While the current literature offers in-depth information on subsets of synapses within the spinal cord, a comprehensive and comparative microscopy-based survey of excitatory synapses throughout the spinal cord, i.e. a synaptic map, may enable us to better understand the functional diversity of spinal neural circuits. Furthermore, the existence of subsynaptic molecular architecture is now discernible with novel microscopy techniques and provides an essential new metric with which to understand synaptic diversity.

Tripartite synapses are comprised of a presynaptic neuron, postsynaptic neuron and glial cell, typically an astrocyte^30,31^. It is thought that most (but not all) synapses are contacted by astrocytes via perisynaptic astrocytic processes, which aid in synapse maintenance, neurotransmitter clearance and recycling^32,33^. Single astrocytes are capable of decoding different neural activity and responding accordingly to modulate the activity of single synapses^34^. Evidence from the brain suggests that astrocytes may preferentially associate with structurally complex synapses^35^, and that astrocyte signalling can modify dendritic spine organisation^36^. Comparable analyses of spinal cord tripartite synapses are lacking, despite evidence of functional glial-neuronal interactions within spinal circuits. For example, astrocytic release of purines modulates synaptic activity of ventral horn interneurons, which in turn regulates the frequency of locomotor-related activity generated by spinal central pattern generator circuits^25,37,38^. Meanwhile, within the dorsal horn, it has been shown that glia elicit calcium transients in response to motor-related activity *in vivo*, and that they play a significant role in dorsal horn sensory processing and pain responses^39,40^.

To better understand neural circuit complexity and diversity in the spinal cord, and the role of astrocytes in modulating neural networks, we have performed an optical investigation of spinal cord synapses and tripartite synapses. We have used a genetically engineered mouse line expressing eGFP fused to endogenous PSD95 to visualise the postsynaptic density (PSD) of excitatory synapses, together with immunolabelling to identify presynaptic boutons and astrocytic contacts. Using a combination of high-resolution microscopy, for large scale synaptic mapping, and super-resolution (SR) microscopy, to interrogate the nanostructure of spinal cord tripartite synapses, we report regional and age-dependent differences in synaptic nanostructure. Furthermore, we provide evidence that astrocytes influence the nanostructural organisation and molecular makeup of spinal cord synapses.

## Results

### High-resolution imaging of PSD95 in the spinal cord

**Figure 1:**
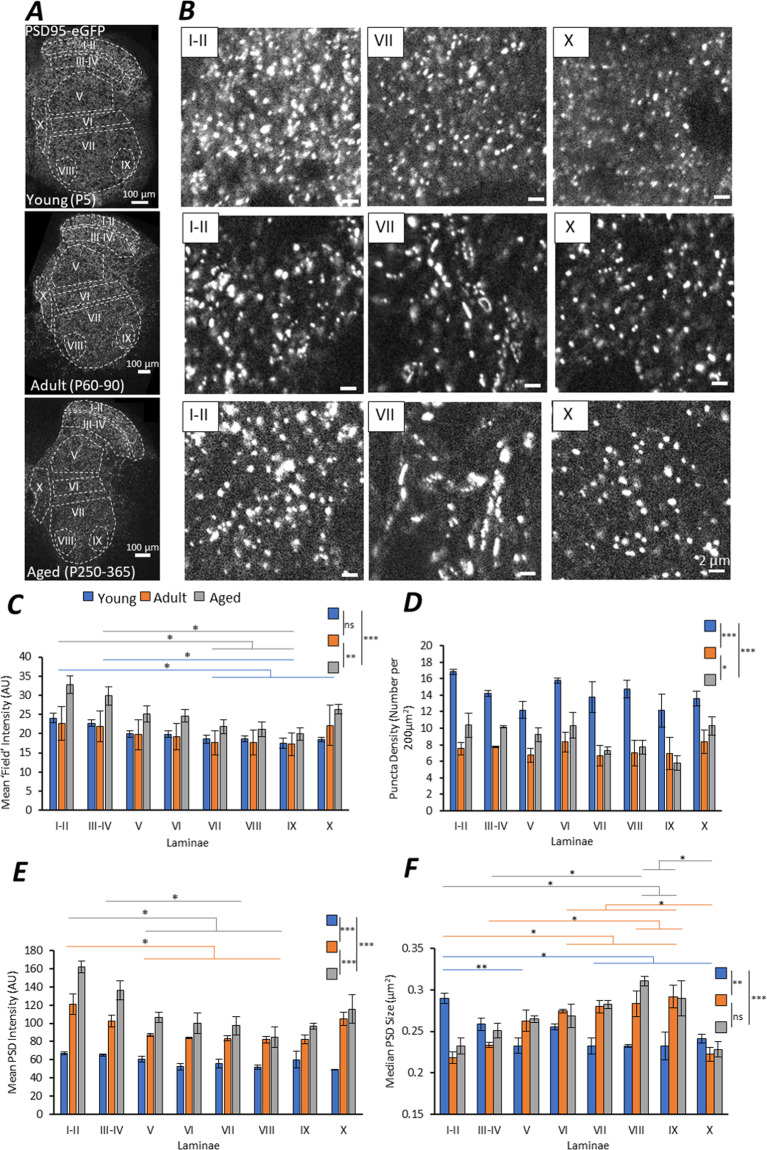
Mapping PSD95-eGFP PSDs in the Mouse Spinal Cord. (**A**) High magnification tiled imaging was performed on half-spinal cord sections from PSD95-eGFP mice from three age groups (n  = 3 mice per age group). Quantitative analysis was performed at the single synapse level to study synaptic diversity between laminae and across age groups. (**B**) Example images of PSD95-eGFP PSDs in three laminae from each age group. Note the formation of large elongated PSDs in VII of Adult and Aged mice. (**C**) The mean ‘field’ intensity of PSD95-eGFP labelling was quantified from entire delineated laminae at each age. Age groups are colour coded as Young (blue), Adult (orange) and Aged (grey). Colour-coded lines indicate statistical significance between laminae within an age group following a post-hoc test. The PSD density (number per unit area) (**D**), the mean fluorescence intensity of PSDs (**E**) and the median PSD size (**F**) is also plotted for each lamina in each age group.

We first performed high-resolution ‘mapping’ of lumbar transverse spinal cord sections from PSD95-eGFP mice to resolve the PSDs of individual, excitatory synapses. We investigated spinal cord PSDs in mice from three different age groups: ‘Young’ neonatal mice (P5); early ‘Adult’ mice (P30-60) and ‘Aged’ adult mice (P260-365). PSD95-eGFP was expressed strongly in the grey matter of the spinal cord in each age group (Fig. 1A). At high resolution, PSD95-eGFP showed a punctate expression pattern in all three of the age groups and in all the surveyed regions (Fig. 1B). Approximately 100,000 synaptic puncta were quantified from each spinal cord section per mouse, per age group; and a range of 1,000 to 20,000 PSDs were detected for each laminae from a single section, facilitating a high level of statistical sampling. Measurements were averaged across two hemi-sections for *n* = 3 mice per age group.

We quantified the overall mean fluorescence intensity (field intensity) from the entirety of each laminae to measure overall PSD95 expression levels; a broad metric that depends on PSD density and intensity within a given region (Fig. 1C). A significant difference in field intensity was found between laminae (F(7,48) = 3.8, p < 0.001) and between ages (F(2,48) = 11.6, p < 0.0001). PSD-95 levels were equivalent in young and adult mice. However, greater levels of PSD-95 were observed in tissue from aged mice compared to young (p < 0.001) or adult (p < 0.0001) animals. Anatomically, there was a greater expression of PSD95-eGFP in the dorsal horn laminae, gradually reducing ventrally. This dorsal-ventral gradient appeared to accentuate with age. Lamina X also showed a particularly high level of PSD95-eGFP expression, most notably in Adult and Aged mice, comparable to dorsal expression levels.

We next performed quantitative analysis of individual PSDs to determine whether there were differences in the number of puncta per unit area (PSD density, Fig. 1D), the mean fluorescence intensity of individual puncta (Fig. 1E) or the size of individual puncta (Fig. 1F) across different laminae and with age. The PSD density measurements significantly differed between ages (Fig. 1D; F(2,48) = 76.454, p < 0.0001), with Young mice showing a significantly higher density of PSDs compared to Adult and Aged mice (both p < 0.0001). Aged mice showed a slightly greater PSD density compared to adult mice (p < 0.05), most notably in laminae I-II, III-IV and V. Overall, the area of the grey matter did not change across the age groups (F(2,48) = 3.02, p  = 0.058). No differences were observed in the PSD density between laminae within either Young, Adult or Aged mice (Fig. 1D; Young: F(7,48) = 1.959, p  = 0.126; Adult: F(7,48) = 0.298, p  = 0.945; Aged: F(7) = 2.997, p  = 0.033 but showed no specific inter-laminal differences at p < 0.05 following a post-hoc Tukey’s Test).

The mean intensity of PSD95-eGFP in PSDs significantly increased with age (Fig. 1E; F(2,48) = 121.67, p < 0.0001). There was a 1.6-fold increase in mean PSD intensity between Young and Adult mice and a 1.2 fold increase between Adult and Aged mice, suggesting progressive enrichment of PSD95 at synapses during development and ageing (Fig. 1E). This age dependent increase in PSD95 was most prominent in laminae I-II, III-IV and X with approximately a 2.4-fold increase in mean fluorescence intensity between Young and Aged mice. Regional differences in PSD mean intensity were observed in adulthood (Fig. 1E), with significant differences between laminae in Adult (F(7,48) = 6.239, p < 0.01) and Aged mice (F(7,48) = 6.19, p < 0.01), but not in Young mice (F(7,48) = 2.479, p = 0.063). PSDs in laminae I-II were most concentrated with PSD95 molecules, compared with PSDs in the medial motor pool (VIII) in both Adult (1.5 fold; p < 0.01) and Aged mice (1.9 fold, p < 0.001), which were the least intensely labelled. PSDs in most laminae showed an incremental increase in PSD95-eGFP mean intensity with age. PSDs in ventral laminae and lamina X, however, showed virtually no change between Adult and Aged mice.

We next investigated whether PSDs differed in size with age or between anatomical regions (Fig. 1F). It was found that PSD size differed significantly with age (F(2,48) = 7.357; p < 0.01) and between laminae (F(7,48) =  6.872, p < 0.0001). Young mice showed significant anatomical diversity in PSD size (F(7,48) = 5.551, p < 0.001), with laminae I-II PSDs being significantly larger than those of laminae V (p < 0.01), VII (p < 0.01) and X (p < 0.05). In Adult and Aged mice however, laminae I-II PSDs were smaller, while PSDs in ventral laminae were much larger. For example, PSDs of lamina VIII were significantly larger than dorsal I-II PSDs in both Adult (p < 0.01) and Aged (p < 0.01) mice. Ventral PSDs often showed more elongated and complex morphologies than in other laminae (Fig. 1B). Interestingly, PSDs in lamina V, VI and X did not show age dependent changes in PSD size, while dorsal laminae (I-II, III-IV) and ventral laminae (VII, VIII, IX) showed opposing changes in size with age.

As a metric of total content of PSD95 per synapse, we calculated the integrated intensity of PSDs, which combines the intensity of each pixel and the size of the segmented PSDs (SI. Figure 1). The integrated intensity of PSDs increased significantly with age (F(2,48) = 200.2; p < 0.0001) and displayed a significant dorsal-to-ventral gradient in both the Young (F(7,48) = 11.2, p < 0.0001) and Aged (F(7,48) = 3.3, p < 0.05) mice. Interestingly, Adult mice showed no difference in the integrated intensity of PSDs between laminae (F(2,48) = 1.2; p < 0.374), which may correspond with the appearance of large PSDs in the ventral laminae in contrast to the smaller but brighter PSD95-eGFP PSDs in the dorsal laminae.

From these large-scale analyses of PSD95-eGFP expression at the single synapse level, we have obtained a quantitative map of excitatory synapse PSDs in the mammalian lumbar spinal cord. Overall, we observe a loss of excitatory PSDs during development, but those that remain are increasingly enriched with PSD95 during ageing. Anatomically we observe a key distinction between dorsal and ventral PSDs in adult mice, whereby dorsal horn PSDs are smaller but contain greater PSD95 content than those in the ventral horn. This anatomical diversity is highly dependent on age, suggesting different developmental changes in synaptic organisation between sensory (dorsal) and motor (ventral) circuits.

### Nanostructural organisation of PSD95 in spinal cord synapses

Having demonstrated differences in the structure and molecular makeup of synapses across different spinal laminae and age groups, we next sought to understand the nanostructural organisation of PSD95 at spinal cord synapses. At a molecular level, PSD95 is known to form nanoclusters (NCs) of approximately 70-200 nm in size within excitatory PSDs^4,5,18,19^. Therefore, we utilised fluorescence-based SR microscopy, specifically gated-stimulated emission depletion (g-STED) microscopy, to investigate the diversity in postsynaptic nanostructure within the spinal cord.

**Figure 2:**
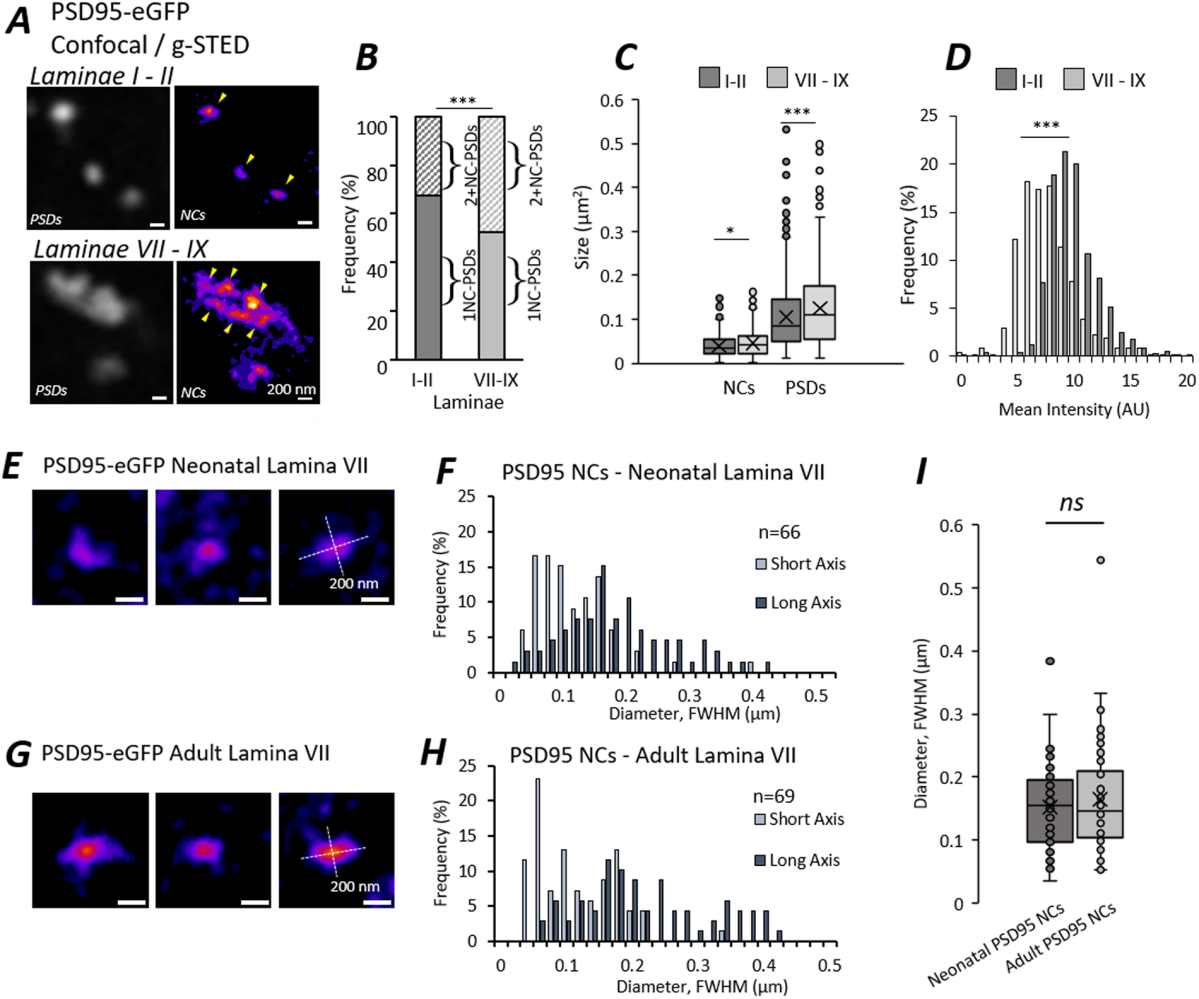
Super-Resolution Microscopy of Postsynaptic Nanostructure in the Mouse Spinal Cord. (**A**) Confocal and g-STED images of PSD95-eGFP were captured from a single adult spinal cord section. Confocal images resolve overall PSDs (n  = 788 PSDs from laminae I-II, n  = 295 PSDs from laminae VII-IX) while g-STED images super-resolve NC substructures (n  = 1151 NCs from laminae I-II, n  = 527 NCs from laminae VII-IX), highlighted with yellow arrowheads. (**B**) Stacked percentage plot of PSDs defined as 1NC-PSDs or 2+NC-PSDs from the two regions. (**C**) Box and whisker plots of the sizes of PSDs and NCs quantified from each region. (**D**) Histogram of the mean fluorescence intensities of PSD95-eGFP NCs measured from each region. **(E**) Example PSD95-eGFP NCs visualised in lamina VII of neonatal mouse spinal cord, with short and long axes denoted for FWHM analysis. (**F**) Histogram plotting the distribution of neonatal PSD95 NC short and long axis lengths from FWHM analysis (n  = 66 NCs). (**G**) Example PSD95-eGFP NCs visualised in lamina VII of adult mouse spinal cord, with short and long axes denoted for FWHM analysis. (**H**) Histogram plotting the distribution of adult PSD95 NC short and long axis lengths from FWHM analysis (n  = 69 NCs). (**I**) Box and whisker plot of the mean diameters of PSD95 NCs from both neonatal and adult mouse spinal cords, showing no significant difference in NC size between age groups.

We visualised PSD95-eGFP by capturing correlative confocal images (~250 nm XY resolution) and g-STED images (~80 nm XY resolution) to resolve and quantify PSDs and NCs respectively (Fig. 2A); a method which has been applied in previous work^4,19^. A total of 788 PSDs and 1151 NCs were quantified from dorsal spinal cord laminae and a total of 295 PSDs and 527 NCs were quantified from ventral horn laminae from 1 adult mouse section.

First, we quantified the number of NCs per PSD, and subsequently compared the number of 1NC-PSDs and 2+NC-PSDs between dorsal and ventral laminae (Fig. 2B). We found that ventral laminae contained a greater proportion of 2+NC-PSDs (47%) than the dorsal laminae (32%) (χ(1) = 21.1, *p* < 0.0001).

Next, we analysed the sizes of PSD95-eGFP PSDs and NCs (Fig. 2C) using correlative confocal and g-STED microscopy. Consistent with the high-resolution imaging, confocal microscopy revealed that PSDs in ventral laminae were significantly larger than those in dorsal laminae (Fig. 2C; U  = 137,415, p < 0.0001). g-STED microscopy revealed that individual NCs in the ventral laminae were also larger than those in the dorsal laminae (Fig. 2C; U  = 291,747, p < 0.05), although this difference was far less prominent than the difference in PSD size between the two laminae. Interestingly, while ventral synapses were typically larger and more complex in their structure, dorsal PSD95 NCs were found to have a significantly greater mean fluorescence intensity than ventral NCs (Fig. 2D; U  = 430,118; p < 0.0001).

These data suggest that structural differences between dorsal and ventral synapses are predominantly determined by the number of NCs per PSD, while molecularly these regions differ in the number of PSD95 scaffolding molecules packed into individual synapse subdomains.

We next asked whether age had an impact on the nanostructural organisation of PSD95 within synapses. g-STED microscopy was performed on young and adult mouse sections in the ventral horn. In order to more accurately quantify the size and shape of PSD95-eGFP NCs in the neonatal (Fig. 2E,F) and adult (Fig. 2G,H) spinal cord, synapses were analysed by taking the full width at half maximum (FWHM) along line profiles depicting the minimum and maximum diameters of individual NCs. NCs that appeared to be alone, indicative of 1 NC-PSDs were elected for analysis to avoid overlap with other nanostructures. We found that NCs were typically elliptical structures indicative of membrane-associated domains, and did not change significantly in their aspect ratio between neonatal and adult spinal cords (neonatal median diameters: 93 nm x 181 nm; adult median diameters: 92 nm × 193 nm; U  = 2006, p  = 0.233). The mean diameters of PSD95 NCs also showed no significant difference between neonatal and adult spinal cords (Fig. 2I; U  = 2414, p  = 0.546).

These results further confirm the notion that PSD95 NCs are relatively conserved in their basic structure between PSDs in different anatomical regions and across development. However, the concentration of PSD95 molecules within these domains, and the number of NCs within a PSD, appears to vary with age and anatomical region.

### Differences in postsynaptic structure between functionally distinct synapses

We next asked whether postsynaptic structure was dependent on the source of presynaptic inputs. Vesicular glutamate transporters 1 and 2 (VGLUT1 and VGLUT2) commonly label distinct populations of presynaptic boutons in different pathways throughout the nervous system^20,27,41–43^. Within the spinal cord, VGLUT1 labels primary mechanoreceptive terminals from dorsal root ganglia sensory neurons, as well as a smaller number of synaptic boutons arising from corticospinal pathways^43,44^. Meanwhile, the majority of VGLUT2 boutons derive from local spinal neurons, with a smaller number arising from nociceptive inputs^43,45^, and descending inputs from the rubrospinal and vestibulospinal tracts^46^. To assess whether postsynaptic structure differed depending on the presence of VGLUT1 versus VGLUT2 at presynaptic terminals, we performed high-resolution and SR microscopy on adult PSD95-eGFP mouse spinal cord sections co-immunolabelled for VGLUT1 or VGLUT2.

**Figure 3:**
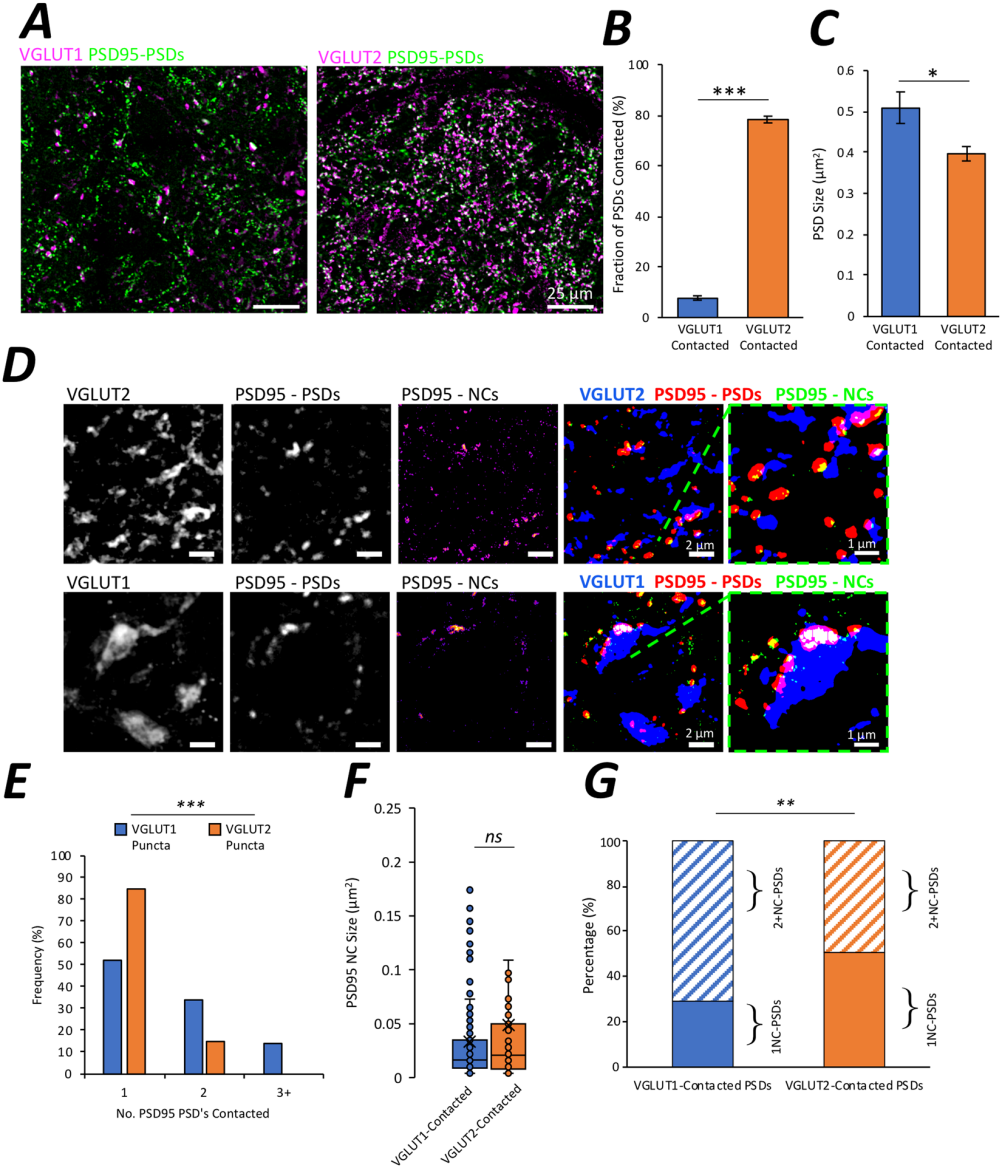
Presynaptic Inputs and Postsynaptic Diversity in the Mouse Spinal Cord. (**A**) High-resolution microscopy reveals the expression of VGLUT1 and VGLUT2 alongside PSD95-eGFP (n  = 5 animals). (**B**) Bar chart depicting the percentage of PSDs contacted by VGLUT1 and VGLUT2 boutons. (**C**) Bar chart depicting the size of PSDs contacted by VGLUT1 and VGLUT2 boutons. (**D**) Super-resolution g-STED microscopy of VGLUT1 and VGLUT2 alongside PSD95-eGFP PSDs and NCs (n  = 27 VGLUT1 boutons contacting n  = 47 PSDs; and n  = 63 VGLUT2 boutons contacting n  = 76 PSDs). Far right-hand panels show thresholded images for analysis of postsynaptic nanostructure. (**E**) Frequency histogram showing the proportion of VGLUT1 and VGLUT2 boutons that contact either 1, 2 or 3 or more PSDs. (**F**) Box and whisker plot displaying the sizes of PSD95 NCs contacted by either VGLUT1 or VGLUT2. (**G**) Stacked bar chart displaying the fractions of 1 NC-PSDs and 2+NC-PSDs for both VGLUT1 and VGLUT2 contacted synapses.

High-resolution microscopy of PSD95-eGFP and immunolabelling for VGLUT1 and VGLUT2 (n  = 5 mice), revealed expected distributions of glutamate transporters. VGLUT1 expression was isolated to more discrete locations in laminae III-IV, V and IX, while VGLUT2 expression was more widely spread across laminae. VGLUT2 boutons were also typically smaller and more numerous than VGLUT1 boutons (Fig. 3A). As such, ~8% of PSD95-eGFP PSDs were juxtaposed with VGLUT1 boutons, while ~78% of PSDs were associated with VGLUT2 boutons (Fig. 3B; t(8)  =  44.5, p < 0.0001). Analysis of the size of PSD95 PSDs revealed that PSDs contacted by VGLUT1 were larger (0.51 μm^2^) than those contacted by VGLUT2 (0.40 μm^2^) (Fig. 3C; t(8) = 2.7, p  = 0.029).

Using g-STED microscopy, we next investigated the nanostructure of these two subtypes of synapses. Images were captured of PSD95 along with either VGLUT1 or VGLUT2 (Fig. 3D). At a nanostructural level, VGLUT2 boutons appeared to be made up of multiple small structures (median diameter 90 nm; n  = 125 VGLUT2 substructures; Fig. 3D; SI. Figure 2A–C). Meanwhile, VGLUT1 boutons displayed a range of nanostructural morphologies from small, defined structures to larger, less clearly defined domains (median diameter of all structures 272 nm; n  = 104 VGLUT1 substructures, Fig. 3D; SI. Figure 2D–F).

Next, we used g-STED to investigate postsynaptic nanostructural differences between VGLUT1 and VGLUT2 synapses. Synapses were pooled for analysis from sections across 2 animals (n  = 27 VGLUT1 boutons contacting n  = 47 PSDs; and n  = 63 VGLUT2 boutons contacting n  = 76 PSDs). We found that large VGLUT1 boutons showed a significantly greater tendency to contact multiple, distinct PSDs as compared to VGLUT2 boutons (Fig. 3E; χ(2) = 21.8, *p* < 0.0001). We observed no significant difference in the size of PSD95 NCs between VGLUT1 and VGLUT2 synapse subtypes (n  = 234 NCs part of VGLUT1 synapses, n  = 129 NCs part of VGLUT2 synapses; Fig. 3F, U  = 16079, p  = 0.303). Instead, PSDs associated with VGLUT1 were more likely to contain multiple NCs compared to PSDs associated with VGLUT2 (Fig. 3G, χ(1) = 7.1, *p*  = 0.008). Indeed, 65% of VGLUT1-contacted PSDs were identified as 2+NC-PSDs compared with 49% of VGLUT2-contacted PSDs.

These findings suggest that the molecular composition of presynaptic terminals, may influence postsynaptic morphology at the nanostructural level, to help ensure that synapses are tuned to the demands of specific neural pathways.

### Postsynaptic structure of tripartite synapses

Astrocytes can influence synaptic activity and modulate motor circuits of the spinal cord^25,37,38,47^. We therefore asked whether spinal synapses that are contacted by astrocytes, termed tripartite synapses, are structurally or molecularly different to synapses that are not tripartite.

The structure and location of spinal astrocytes was assessed using immunolabelling for excitatory amino acid transporter 2 (EAAT2), phosphorylated Ezrin (p-Ezrin) and glial fibrillary associated protein (GFAP). GFAP immunolabelling revealed extensive astrocytic arborisations, while EAAT2 immunolabelling appeared as puncta that were mostly aligned with GFAP processes (SI. Figure 3). Many EAAT2 puncta did not colocalise with GFAP immunolabelling, in accordance with reports that EAAT2 is expressed in finer astrocytic processes that express less GFAP^48^. As an additional marker, p-Ezrin was used as it has been shown to label the fine branches of astrocytes which contact synapses^32,49^.

**Figure 4:**
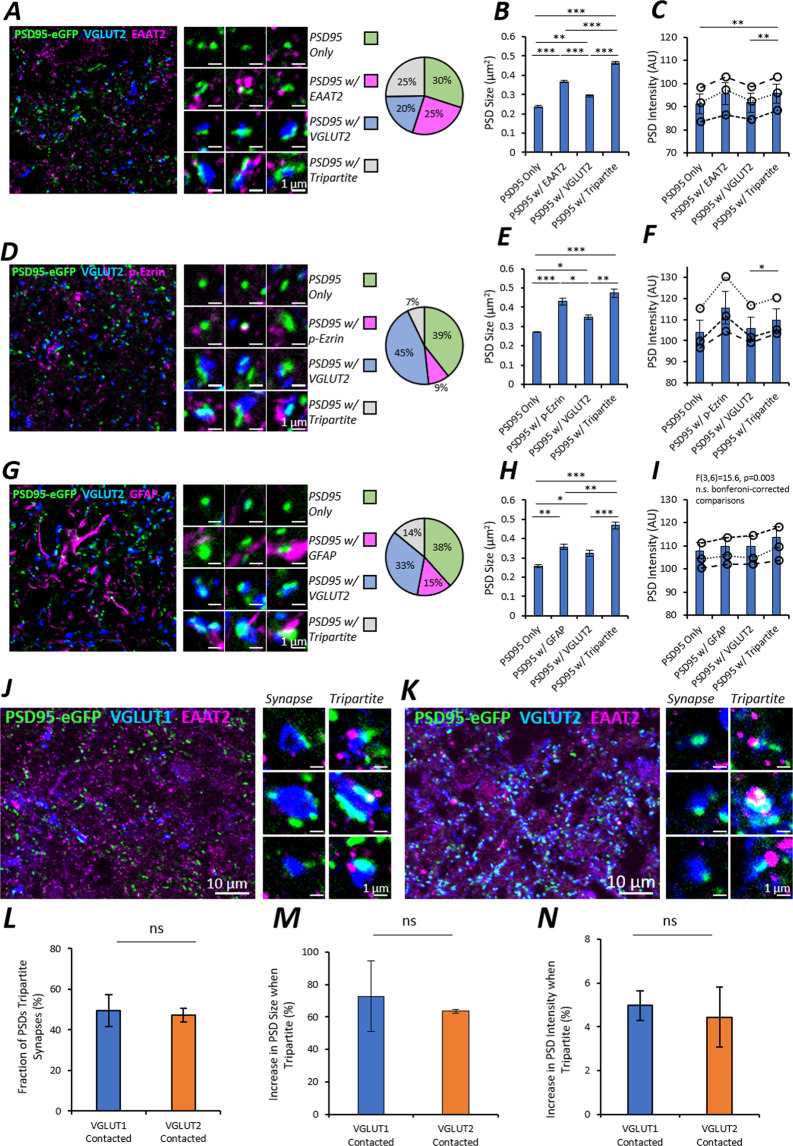
Visualising Tripartite Synapses in the Mouse Spinal Cord. (**A–C**) PSD95-eGFP spinal cord sections (n  = 3 animals) were immunolabelled for VGLUT2 and either EAAT2 (**A**), p-Ezrin (**D**) or GFAP (**G**) to reveal tripartite synapses. In each case, PSD95-eGFP PSDs are categorised into four different groups: PSD95 only PSDs, PSD95 with astrocytic label, PSD95 with VGLUT2, and tripartite synapses with both VGLUT2 and an astrocyte label. (**B,E,H**) Bar charts depicting the sizes of PSD95 PSD subtypes as quantified for each astrocytic marker: EAAT2 (**B**), p-Ezrin (**E**) and GFAP (**H**), and analysed with a One-way ANOVA and post-hoc Tukey’s test. (**C,F,I**) Bar charts depicting the fluorescence intensities of PSD95 PSD subtypes as quantified for each astrocytic marker: EAAT2 (**C**), p-Ezrin (**F**) and GFAP (**I**). The dashed lines in each bar chart for intensity depict the measurements from each mouse with statistics performed as a repeated measures ANOVA to account for baseline variance between mice. (**J,K**) In a separate set of experiments, PSD95-eGFP sections (n  = 5 animals) were immunolabelled for EAAT2 and either VGLUT1 (**J**) or VGLUT2 (**K**) to study the effect of astrocytes on functionally distinct synapses. Images were captured from the ventral horn laminae VII-IX. (**L**) Bar chart comparing the fraction of VGLUT1- and VGLUT2-contacted synapses that were tripartite. (**M,N**) Bar charts comparing the % increase in PSD size (**M**) and PSD95-eGFP intensity (**N**) when synapses are contacted by astrocytic EAAT2 between VGLUT2 and VGLUT1-associated synapses.

Tripartite synapses were visualised in ventral laminae VII-IX of adult PSD95-eGFP mouse sections using high-resolution microscopy (n  = 3 adult mice). Immunolabelling for presynaptic boutons (VGLUT2) and astrocytes (EAAT2, p-Ezrin or GFAP) was performed to identify the components of tripartite synapses (Fig. 4A–I). PSD95-eGFP PSDs were categorised into four groups based on their colocalisation with astrocytic and presynaptic components: PSD95 only, PSD95 with an astrocytic marker, PSD95 with VGLUT2 (classified here as a synapse) and PSD95 with both an astrocytic marker and VGLUT2 (classified here as a tripartite synapse) (Fig. 4A,D,G). We observed that 25% of the total number of PSD95-eGFP PSDs (56% of synapses, as defined by their VGLUT2 apposition) were tripartite based on their association with EAAT2. We also observed that 7% of the total number of PSDs (14% of synapses) were contacted by p-Ezrin, while 14% of PSDs (30% of synapses) were contacted by GFAP.

PSDs that were part of a tripartite synapse were observed to be 50–60% larger in size (Fig. 4B,E,H) and 4–5% brighter in PSD95-eGFP intensity within the PSD (Fig. 4C,F,I). This held true when using EAAT2 (size: F(3) =  224.7, p < 0.0001; intensity: F(3,6) = 35.0, p < 0.0001) and p-Ezrin (size: F(3) =  33.3, p < 0.0001; intensity: F(3,6) = 17.1, p < 0.01), and to a lesser extent this effect was observed with GFAP as the astrocytic marker (size: F(3) =  38.0, p < 0.0001; intensity: F(3,6) = 15.6, p < 0.01, though no pairwise differences were observed following Bonferroni-corrected comparisons).

**Figure 5:**
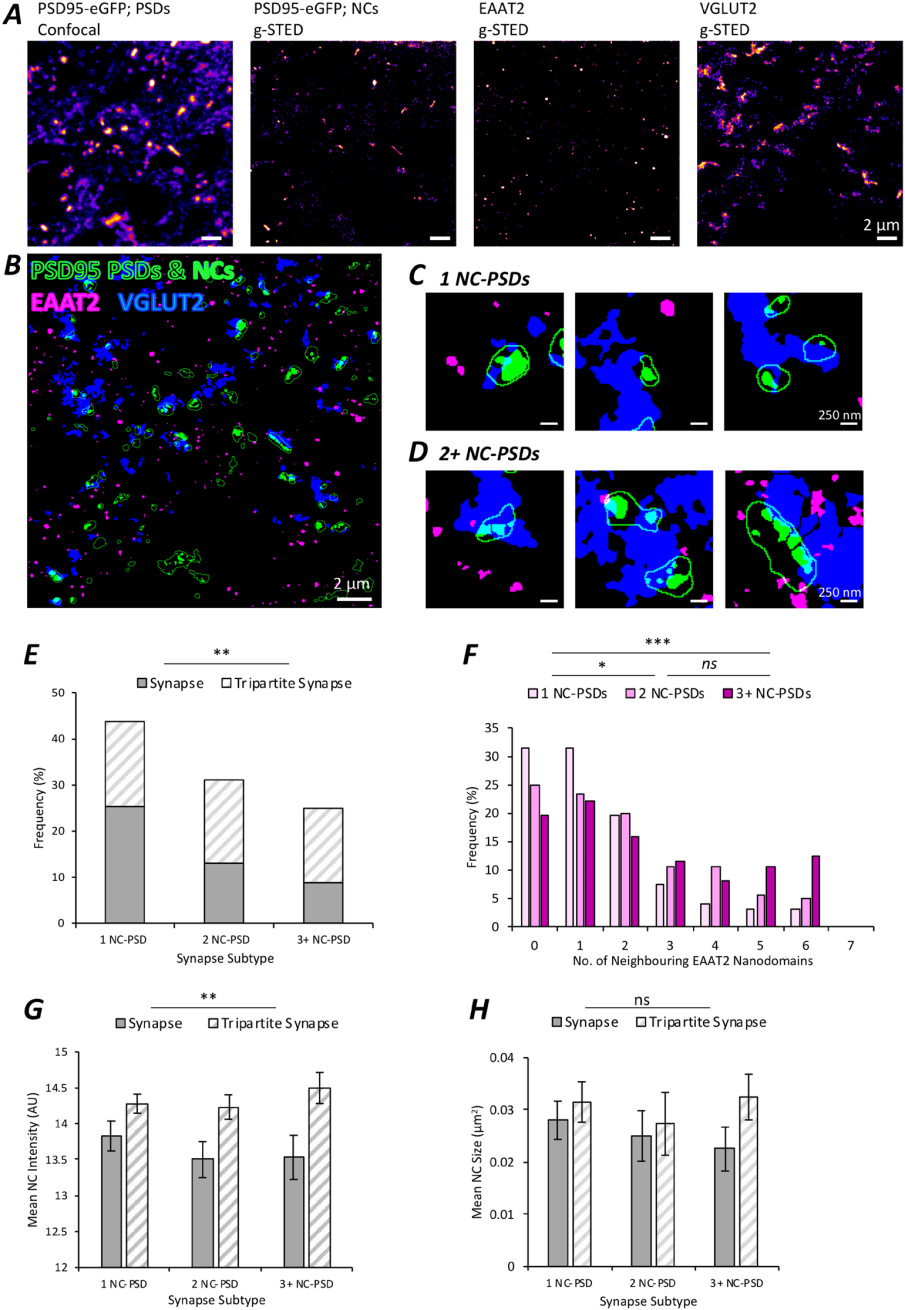
Tripartite Synapse Nanostructure in the Mouse Spinal Cord. (**A**) Confocal microscopy of PSD95-eGFP was performed in adult mouse spinal cord sections to visualise PSDs (n  = 452 PSDs). PSD95 NCs were visualised using correlative g-STED microscopy (n  = 950 NCs). EAAT2 nanodomains and VGLUT2 presynaptic boutons were also visualised with g-STED microscopy. (**B**) Images were processed and thresholded to reveal the PSDs (green outlines), PSD95 NCs (green filled clusters) within the green outlined PSDs, presynaptic terminals (VGLUT2 in blue), and astrocytic contacts (EAAT2 nanodomains in magenta). (**C**) Examples of simple 1 NC-PSDs. (**D**) Examples of complex 2+ NC-PSDs. (**E**) A stacked bar chart showing the fractions of synapses that are tripartite (striped grey) versus synapses not contacted by astrocytes (grey fill), subclassified as to whether the PSD contained 1 NC, 2 NCs or 3+ NCs. (**F**) A frequency histogram comparing the proportions of 1 NC-PSDs, 2 NC-PSDs and 3+ NC-PSDs by the number of astrocytic EAAT2 nanodomains residing within 200 nm of a PSD (edge-edge distance). (**G**) Bar chart displaying the mean fluorescence intensity of PSD95-eGFP NCs, classified by whether the synapse was tripartite or not contacted by astrocytes, and whether the PSD contained 1 NC, 2 NCs or 3+ NCs. (**H**) Bar chart displaying the mean fluorescence intensity of PSD95-eGFP NCs, classified by whether the synapse was tripartite or not contacted by astrocytes, and whether the PSD contained 1 NC, 2 NCs or 3+ NCs.

In additional analyses, EAAT2 structures that were the ‘nearest neighbours’ to each PSD95-eGFP puncta were detected (SI. Figure 4A-E). As a control for random association of objects, the same analysis was performed when the EAAT2 images were rotated 180° from their original orientation (SI. Figure 4B). We observed that the average number of EAAT2 puncta within a 3 µm radius of each PSD95-eGFP PSD was greater in the true images as compared with the rotated controls (SI. Figure 3C; t(2) = 8.4, p < 0.05). Similarly, the average distance between PSDs and the nearest 10 EAAT2 particles was significantly less in the true images as compared to the rotated controls (SI. Figure 4D; t(2) = 8.1, p < 0.05). Finally, using nearest neighbour analysis, we confirmed that PSD95-eGFP PSDs are significantly larger when there are a greater number of neighbouring EAAT2 particles (SI. Figure 4E; t(4) = 2.8, p < 0.05), an effect which is not observed in the rotated control images (t(4) = 1.1, p  = 0.322). From these nearest neighbour analyses, the results suggest that the association of astrocytic EAAT2 with synapses is not random and is linked to changes in synapses.

We next asked whether VGLUT1 and VGLUT2 synapses within ventral laminae VII-IX are equally associated with astrocytes (assessed using EAAT2 labelling; n  = 5 mice; Fig. 4J-K). We found that similar proportions of PSDs were part of tripartite synapses, whether presynaptic terminals were marked by VGLUT1 or VGLUT2 expression, suggesting that both synapse subtypes were just as likely to be contacted by astrocytes (Fig. 4L; t(8) = 0.24, p  = 0.816). The effect of astrocytes on PSD size was also found to be no different between VGLUT1 and VGLUT2 associated synapses (Fig. 4M; t(8) = 0.42, p  = 0.686). Similarly, the effect of astrocytes on PSD intensity was also found to be no different between VGLUT1 and VGLUT2 associated synapses (Fig. 4N; t(8) = 0.35, p  = 0.737).

Our data suggest that astrocytes contact different subtypes of excitatory synapses in equal measure. Moreover, the presence of astrocytes at spinal synapses may help to increase the size of the PSD and increase PSD95 levels at the PSD, an effect which appears to be conserved across VGLUT1 and VGLUT2 synapse subtypes.

### Postsynaptic nanostructural complexity of tripartite synapses

Having shown that PSDs are larger and brighter when part of a tripartite synapse, we next interrogated the nanostructural organisation of tripartite synapses. We employed g-STED to super-resolve PSD95-eGFP along with VGLUT2 and EAAT2 (Fig. 5A–D). Analysis was performed on a total of 452 synapses, using images captured from spinal cord sections from 2 different animals. EAAT2 formed very small, uniform domains with a median diameter of 119 nm, and very high aspect ratio – indicative of small circular clusters (SI. Figure 2G-I). Our analysis approach enabled the quantification of the number of NCs per PSD, whether EAAT2 domains colocalized with the PSD, as well as the number of neighbouring EAAT2 domains within 200 nm of the boundary of a PSD.

We observed that synapses were significantly more likely to be tripartite when the PSD was comprised of more NCs (Fig. 5E; χ(2) = 12.1, *p*  = 0.002). In addition, 2NC-PSDs and 3+ NC-PSDs had a significantly greater number of individual EAAT2 domains within 200 nm distance from the PSD, compared to 1NC-PSDs (Fig. 5F; χ(2) = 20.8, *p* < 0.0001). These results confirm that large multi-NC-PSDs are more likely to be part of a tripartite synapse.

Next, we assessed whether PSD95 NCs differed in their size or in their concentration of PSD95 molecules (mean fluorescence intensity) depending on their tripartite status. We found that tripartite synapses were more likely to have NCs that were brighter and were therefore more enriched with PSD95 (Fig. 5G; F(1,446) = 10.0, p  = 0.002). There was, however, no difference in the fluorescence intensity of PSD95-eGFP NCs between different nanostructural subclasses of PSDs (Fig. 5G; F(2,446) = 1.4; p  = 0.259). In addition, we found no difference in the sizes of PSD95 NCs whether they were part of a tripartite synapse or not (Fig. 5H, F(1,446) = 0.80, p  = 0.371), or whether they belonged to a 1 NC-PSD, 2 NC-PSD or 3+ NC PSD synapse (Fig. 5H, F(2,446) = 0.65, p  = 0.524).

Together, these data show for the first time that the presence of astrocytes at synapses is associated with increased nanostructural complexity of the PSD. This greater complexity reflects an increase in the number of scaffolding domains within the PSD, as well as an enrichment in the molecular content (increased PSD95 expression) of individual domains within the PSD.

### Astrocytic activity alters PSD composition

Having established that tripartite synapses are more nanostructurally complex and are enriched with the postsynaptic scaffolding protein PSD95, we next, investigated whether astrocytic activity is responsible for evoking structural and molecular changes in the PSD, or whether astrocytes preferentially contact synapses that are already ‘stronger’.

**Figure 6:**
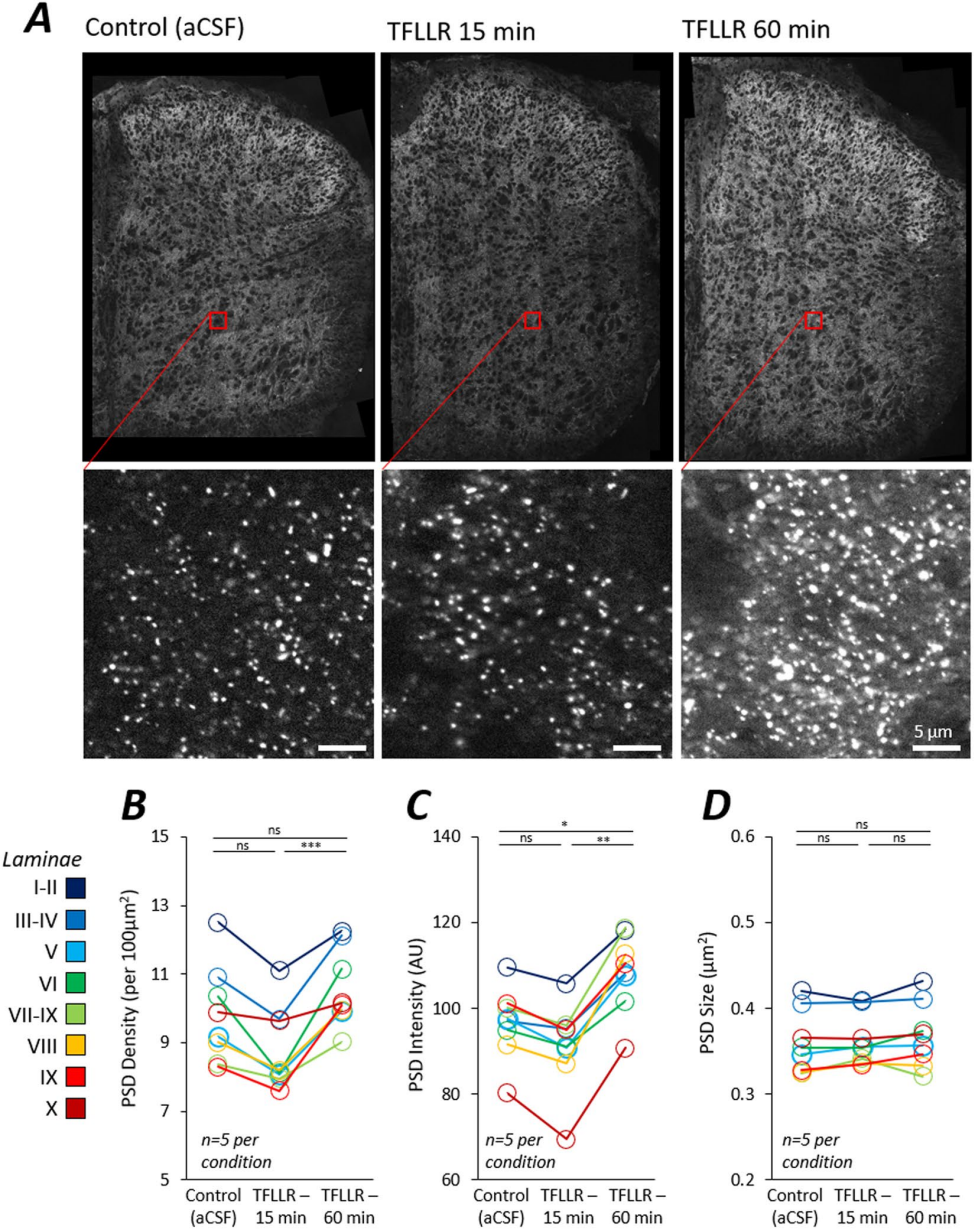
Astrocyte Stimulation using PAR1 Agonist Induces Postsynaptic Alterations. (**A**) Neonatal mouse spinal cords were incubated in aCSF (Control), TFLLR-15 min or TFLLR-60 min (n  = 5 per condition). Spinal cords were subsequently fixed, processed and visualised with high-resolution mapping microscopy. Cropped images show PSD95-eGFP puncta indicative of PSDs. (**B**) Plot of PSD density in response to the three treatment conditions for each colour coded lamina. (**C**) Plot of PSD intensity in response to the three treatment conditions for each colour coded lamina. (**D**) Plot of PSD size in response to the three treatment conditions for each colour coded lamina.

To address this question, we pharmacologically stimulated astrocytes in isolated neonatal spinal cords using a ligand for the astrocyte-specific protease-activated receptor, PAR1 (TFLLR, 10 μM). Isolated spinal cords were incubated in aCSF for a total of 90 minutes before being chemically fixed and cryosectioned for post-hoc analyses of PSDs (Fig. 6A). In our three experimental conditions, TFLLR was either absent throughout (control), applied for the final 15 minutes, or applied for the final 60 minutes (n  = 5 mice per condition).

Overall, pharmacological activation of astrocytes evoked significant changes in PSD density (Fig. 6B; F(2,96) = 7.06, p < 0.01) and intensity (Fig. 6C; F(2,96) = 6.13, p < 0.01) but not PSD size (Fig. 6D; F(2,96) = 1.47, p  = 0.24). Following brief activation with TFLLR for 15 mins, PSD density was found to be reduced slightly (though not significantly) compared to controls (Fig. 6B; p  = 0.091), while prolonged application of TFLLR for 60 mins resulted in significantly higher PSD density compared to the 15 min group (Fig. 6B; p < 0.001). Brief activation of astrocytes for 15 mins had no significant effect on PSD intensity (Fig. 6C*;* p  = 0.56). But following prolonged 60 mins activation, PSD intensity was significantly greater than both controls (p < 0.05) and the 15 min condition (p < 0.01).

These data show that elevated astrocytic activity leads to alterations in postsynaptic molecular composition and thus support the notion that astrocytic signalling may play a role in determining synaptic structural complexity. The effect of astrocytic stimulation on PSD parameters was independent of the anatomical laminae, suggesting that functionally and molecularly different synapses within different regions and circuits of the spinal cord are all equally affected by astrocytic signalling.

## Discussion

This study provides a highly quantitative microscopy-based investigation of excitatory tripartite synapses in the mammalian spinal cord using a genetically engineered mouse model with both high-resolution and super-resolution microscopy methods. These data reveal a host of novel findings regarding postsynaptic diversity across functionally distinct laminae, development and ageing and different neural pathways. Furthermore, we provide evidence that astrocytes help to increase molecular content at the PSD and may aid in the formation of complex postsynaptic morphologies.

Currently there is no single, standardised method to accurately visualise and quantify tripartite synapses. While EM is regarded as one of the most accurate approaches to determine such complex and minute structures^35^, it is not practical for large-scale quantitative studies^50^. Fluorescence-based microscopy enables the generation of large and readily quantifiable data sets. In addition novel super-resolution imaging techniques now enable the dissection of components of tripartite synapses at subsynaptic-level resolution^50,51^.

The genetically engineered PSD95-eGFP mouse readily enables quantitative analysis of excitatory synapse structure and relative abundance of PSD95 using numerous forms of microscopy^4,20^. As one of the most abundant scaffolding proteins of the PSD^12,52,53^, PSD95 provides a robust and reliable marker for excitatory synapses. Similarly, VGLUT1 and VGLUT2 label the majority of excitatory presynaptic boutons^43^. Thus, by combining PSD95 and VGLUT labelling, we are confident that our approach has enabled a relatively comprehensive analysis of excitatory synapses throughout the mammalian spinal cord.

We labelled astrocytes using antibodies raised against EAAT2, p-Ezrin and GFAP. Identifying a single molecular marker to label astrocytic processes reliably and accurately is technically challenging. While GFAP is a canonical label for astrocytes, it may not label all astrocytes, nor the most distal processes of astrocytes^54–56^. EAAT2 and p-Ezrin are more frequently associated with the perisynaptic astrocytic process nearby synapses^49,57,58^, with EAAT2 showing little-to-no neuronal expression in the postnatal mouse spinal cord^59^. While we elected to focus on EAAT2 as a robust marker for perisynaptic astrocytic processes, other markers such as Ezrin (as opposed to p-Ezrin), Glutamine Synthetase or metabotropic Glutamate Receptors may also provide reliable astrocytic labelling for studying tripartite synapses^32,49^. Alternatively, the use of fluorescent reporter mouse lines could be used in combination with our PSD95-eGFP mouse model to interrogate tripartite synapses without the use of immunolabelling^50^. However, such genetic strategies can be affected by incomplete penetrance, which in our case would lead to fewer astrocytes being successfully labelled^60^. In our study we identified that approximately 56% of synapses were tripartite as defined by being contacted by EAAT2, while p-Ezrin and GFAP appeared to contact a much smaller fraction of synapses (14% and 30% respectively). Lavialle *et al*., reported that approximately 58% of synapses were contacted by Ezrin in the rat hippocampus^49^. Furthermore, ultrastructural studies in the brain typically report slightly larger fractions of PSDs being contacted by astrocytes (~70%)^35,61^. These differences may highlight molecular diversity in the expression levels of certain markers of astrocytic processes between the different regions of the nervous system investigated. Furthermore, the stringent criteria we used for colocalization in our quantitative analyses may result in the exclusion of a number of “true” tripartite synapses.

We revealed a number of examples of diversity across synapses in the spinal cord, including 1) an apparent reduction in the density of PSDs during development, 2) an increase in PSD95 expression levels with age, 3) a structural and molecular distinction between synapses of sensory (dorsal) and motor (ventral) regions and 4) distinct postsynaptic signatures of synapses with different presynaptic inputs. These findings are consistent with results from high-resolution PSD95 expression studies conducted in the brain, showing considerable synaptic diversity across anatomical regions and during ageing^20^.

We found that the density of PSD95-eGFP PSDs almost halved between young and adult mice. The apparent loss of PSDs from neonatal stage to adulthood in the spinal cord is supported by similar studies showing a loss of VGLUT1 and VGAT boutons in early development in the mouse spinal cord^62^, and laminae-specific changes in the density of serotonergic boutons in the developing and ageing chicken spinal cord^63^. One possible explanation for this could be that some small PSD95-eGFP puncta are lost due to synaptic pruning over development if they do not develop into functional synapses, as we also observed a slight increase in the fraction of PSDs contacted by presynaptic boutons between Young and Adult mice. Changes in the neuropil volume over age could also result in changes in synapse density, however, we did not observe considerable changes in the total area of the gey matter between ages. Intriguingly, following an initial developmental reduction in PSD95 PSDs, we observed a slight increase in the density of dorsal horn PSDs in 6–9 month-old mice compared to 2-month-old adult mice, similar to that which is reported in the chicken spinal cord^63^. In comparison, ventral regions showed little change in synapse density over adulthood, which may suggest a greater degree of synaptic turnover in sensory circuits versus motor circuits.

We also found that the total content of PSD95 within PSDs continued to increase throughout development into adulthood. The use of the PSD95-eGFP knockin mouse model renders these measurements robust as fluorescence intensity should linearly correlate with endogenous protein content at single synapses^64^. Interestingly, while the expression levels of PSD95 in synapses of dorsal laminae appeared to increase during both development and ageing, levels appeared to remain relatively constant in ventral laminae from adulthood onwards. This suggests that synapses within motor circuits may stabilise by adulthood, whilst synapses within sensory circuits may continue to adapt throughout ageing. Knock-down or inhibition of PSD95 has been shown to reduce somatosensory sensitisation and hyperalgesia^65^, attenuate inflammatory pain^66^ and prevent the induction of neuropathic pain^67–69^; all of which are coordinated by dorsal horn sensory circuitry. It is conceivable that the high levels of PSD95 expression we report within dorsal synapses render these circuits susceptible to dysfunction and disease.

Comparing synapse morphology between young and adult mice also revealed considerable alterations in postsynaptic structure. Large complex PSDs were observed frequently in the adult ventral horn, while young neonatal mice displayed only small and simple PSDs in ventral regions, suggesting a motor-specific and age-dependent change in synapse structure. It is conceivable that further dissection of the excitatory synapse map of the spinal cord between P5 and P30 may reveal a correlation between synapse complexity and the emergence of motor behaviours, such as the onset of weight-bearing (P8–10) or initiation of adult-like walking (P14-16)^70,71^.

PSD95 forms distinct nanostructural domains within synapses. We have termed these subsynaptic PSD95 structures nanoclusters (NCs), though they have also been referred to as nanodomains or subdomains^18,19^. Previous studies indicate that these PSD95 NCs support neurotransmitter receptors^5,18^ and align with presynaptic vesicle release sites enriched with Rab3-interacting molecule (RIM)1/2 subdomains^1^. Levels of both RIM1/2 and PSD95 at the synapse are highly correlated with each other and there is an equal proportion of PSD95 NCs to RIM1/2 subdomains^1^. Thus, based on the literature, we hypothesise that each PSD95 NC visualised within a spinal cord synapse is indicative of the presence of a single trans-synaptic nanocolumn. This nanoarchitecture is thought to enable action-potential-evoked vesicular release at directly opposing postsynaptic receptor domains for increased synaptic efficiency^1^. It is believed that the size of the synapse, as determined by the number of NCs and associated presynaptic release sites, is correlated with the strength of the synapse^72^. From the findings of others^4,5,18,19,73^, and the super-resolution analysis presented here, a set of rules begin to emerge regarding the nanostructural organisation of the PSD: 1) PSDs vary in the number of NCs they contain; 2) the size and morphology of NCs is relatively conserved across different regions, ages, and irrespective of any association with astrocytes; 3) the concentration of PSD95 molecules within NCs can vary. Similar fundamental principles of the molecular organisation of synapses have also been observed in inhibitory synapses^74,75^. Understanding how synaptic diversity occurs across the nervous system will help us to better understand the functional significance of synaptic nanostructure in the context of neural networks and behaviour.

Based on these emerging ‘rules’ of synaptic diversity, in adults we find that synapses in the dorsal horn and near the central canal show a preference for 1-NC-PSD synapses that are densely packed with PSD95 molecules. Meanwhile ventral horn synapses show a greater tendency for large 2+NC-PSDs with individual NCs showing a reduced concentration of PSD95 molecules. These larger multi-NC-PSDs may correspond to L-type synapses, described in EM-based investigations as large asymmetric synaptic boutons with long appositions (>4μm)^76–78^. Given previous evidence that larger PSDs facilitate increased synaptic strength and stability^1,5,18,79^, our data suggest that ventral horn motor circuits may require stronger, more stable 2+NC-PSD synapses, while sensory circuits of the dorsal horn may utilise 1-NC-PSD synapses that are highly concentrated with PSD95 molecules and perhaps exhibit a greater potential for undergoing functional plasticity.

These rules governing synaptic nanoarchitecture may also explain the postsynaptic differences between VGLUT1 and VGLUT2 synapses. Spinal VGLUT1 synapses are associated with low threshold mechanoreceptive afferents and descending corticospinal inputs, while VGLUT2 synapses are predominantly derived from intrinsic spinal interneurons, with some additional expression amongst high-threshold nociceptive afferents and some descending rubrospinal and vestibulospinal inputs^27,28,43,46,80^. Our structural analyses reveal that VGLUT1 boutons were larger than VGLUT2 boutons, and often contacted multiple distinct PSDs, indicative of Type II glomeruli that are widely reported in the dorsal horn laminae III-IV and associated with both VGLUT1 and VGLUT3 expression^29,81,82^. Additionally, we now show that the PSDs associated with VGLUT1 are typically larger and contain a greater number of PSD95 NCs than those associated with VGLUT2, which would tentatively suggest that VGLUT1 synapses form stronger connections^72,73^. From studies of cell cultures and other CNS regions, VGLUT1 synapses exhibit a lower initial probability of release, are associated with larger amplitude postsynaptic potentials and show synaptic facilitation, while VGLUT2 synapses exhibit a higher probability of release, transmit signals with a greater fidelity and show synaptic depression^83–87^. This may reflect a requirement for particularly robust and reliable synaptic transmission at synapses formed by primary sensory afferents mediating reflexive movements. In other brain regions, however, VGLUT1 synapses are smaller than neighbouring VGLUT2 synapses. Therefore, the functional specifications of these two molecularly distinct synapse subtypes may not be conserved between different regions throughout the nervous system. Nevertheless, our findings that VGLUT1 and VGLUT2-associated spinal cord pathways display synaptic structural diversity parallels recent reports of differences between synapses in thalamic circuits that are distinguished by VGLUT1 and VGLUT2 expression^20^. Together these data support that differential molecular composition of synapses may play a role in determining the synaptic nanostructural organisation and functional specification of distinct neural pathways.

It is important to consider that PSD95 NCs may not be static within the PSD. Indeed it has been shown that NCs display dynamic changes in their structure and number per PSD on the timescale of minutes^5,18,74,88^. Therefore, while synapses appear to be organised by a modular molecular arrangement, the modules and the proteins within the modules undergo dynamic redistribution^73,89,90^.

One of the key findings of our study is that synapses residing near astrocytes have structurally larger PSDs, are typically comprised of more NC units, and are molecularly enriched in terms of the number of PSD95 molecules they contain. Our results corroborate previous EM-based studies which suggest astrocytes are more frequently associated with large ‘perforated PSDs’^35^. From histological data alone, it is difficult to infer whether synapses are in fact being structurally altered by the presence of an astrocytic contact, or whether astrocytes preferentially contact larger synapses. We therefore utilised pharmacological stimulation of astrocytes in live, isolated spinal cord preparations to assess whether the molecular components and structure of synapses can be directly regulated by astrocytic activity. We targeted the PAR1 receptor, which is selectively expressed by glial cells throughout the nervous system^91^, including the spinal cord where it has been used to selectively stimulate astrocytes^37,47^. Our results show that prolonged activation of astrocytes results in increased PSD95 expression within PSDs. Interestingly, while there was little effect on synapses when astrocytes were stimulated for a brief period, there was a slight trend for reduced PSD95 expression, suggesting a bimodal effect of astrocyte stimulation. Stimulation of PAR1 within the spinal cord, using the specific agonist TFLLR, is known to increase extracellular adenosine due to astrocytic release of its precursor ATP^92^. Adenosine acts presynaptically to reduce vesicular release at both inhibitory and excitatory synapses on spinal cord interneurons^38^. Additionally, PAR1 activation has been shown to lead to altered astrocyte structure and increased glutamate clearance from the synaptic cleft, which would also lead to reduced postsynaptic activity^93^. It is possible that brief astrocytic activation and inhibition of synaptic activity may result in an immediate withdrawal of postsynaptic scaffolding molecules from the synapse, whilst prolonged synaptic inhibition may lead to homeostatic synaptic scaling, involving increases in the levels of PSD95 and glutamatergic receptors^5,94,95^. This possible mechanism highlights a means by which signalling at the tripartite synapse could result in activity dependent synaptic plasticity of neural circuits. Although further work will be required to define the exact mechanisms involved, our findings support that astrocytes are not simply associated with larger, brighter synapses, but that astrocytic activity can in fact modulate postsynaptic molecular composition.

Our results provide new insights relevant to a range of fields, from synapse and astrocyte biology to spinal cord physiology. Moving forward, this highly quantitative approach could be employed to answer a variety of important questions regarding the relationship between synaptic structure and network function in health and disease. For example, neurodegenerative motor disorders could be interrogated, such as Amyotrophic Lateral Sclerosis (ALS), which is known to involve both synaptic perturbation and glial-dysfunction^23,96–98^.

## Methods

### Harvesting PSD95-eGFP spinal cord tissue

All experimental procedures were conducted in accordance with the UK Home Office regulations and approved by the University of St Andrews Animal Welfare and Ethics Committee. The PSD95-eGFP mice were obtained through collaboration with Prof Seth Grant and have been used in previous publications^4,20^. The gene targeting strategy for tagging PSD95 has been described previously^15^. Briefly, the enhanced green fluorescent protein (eGFP) protein is fused to the C-terminus of PSD95 by inserting the gene into the open reading frame in the 3′-end before the stop codon of exon 19. Homozygous PSD95-eGFP adult male mice were injected with euthatal (Merial Animal Health) prior to perfusion with 1x phosphate buffered saline (PBS) and then 4% paraformaldehyde (PFA). The spinal cords were subsequently dissected out and post fixed in 4% PFA for another 3 hours before being submerged in 30% sucrose solution (w/v) for two days at 4 °C. Neonatal mice were cervically dislocated and decapitated before spinal cords were dissected free and immediately fixed in 4% PFA for 3 hours before incubation in sucrose. Spinal cords were embedded in Optimal Cutting Temperature (OCT) compound and frozen in blocks for cryosectioning. Upper lumbar segments (L1-L3) were cryosectioned to produce 15 µm thick (± 5 µm) transverse slices.

### Immunohistochemistry

Immunohistochemistry was performed by first blocking the tissue in 3% Bovine Serum Albumin (BSA) and 0.2% Triton X-100 in PBS before incubating in primary antibody solution (1.5% BSA and 0.1% Triton X100) overnight at 4 °C. Primary antibodies used were: rabbit anti-Glial fibrillary associated protein (GFAP; DAKO, Z033429-2; 1:500 dilution); chicken anti-GFAP (Aves, GFAP; 1:500 dilution); mouse anti-Vesicular Glutamate Transporter (VGLUT2; Abcam, ab79157; 1:500); guinea pig anti-VGLUT1 (Millipore, ab5905; 1:500 dilution) rabbit anti-excitatory amino acid transporter 2 (EAAT2; Stratech, bs1751R-BSS; 1:250), and rabbit anti-p-Ezrin (Abcam, ab47293, 1:250). Subsequent washes were performed in PBS before and after secondary incubation. Secondary antibodies used include: donkey anti-rabbit Cy3 (1:500); donkey anti-rabbit Alexa Fluor 594 (1:500), donkey anti-mouse Alexa Fluor 647 nm (1:250) and goat anti-guinea pig (1:250). A final wash in deionised water was conducted prior to the mounting of slices with a coverslip. Vectashield (Vector Labs) was used for high-resolution microscopy, while lab-made Mowiol with DABCO was used for g-STED Microscopy.

### Pharmacological experiments

To assess the effect of glial cell activation on synapse parameters, spinal cords from litter-matched neonatal 5 day old homozygous PSD95-eGFP mice were obtained by dissection in artificial cerebrospinal fluid (aCSF, ~4 °C, equilibrated with 95% oxygen, 5% carbon dioxide) containing (in mM): 127 NaCl, 26 NaHCO_3_, 10 glucose, 3 KCl, 2 CaCl_2_, 1.25 NaH_2_PO_4_, and 1 MgCl_2_. Isolated spinal cords were then kept in separate baths containing aCSF at room temperature, equilibrated with 95% oxygen, 5% carbon dioxide, for a total of 90 mins. The PAR1 receptor specific agonist, TFLLR (10 µM; Sigma-Aldrich), was bath-applied to a subset of spinal cords for either 15 mins or 60 mins prior to their fixation in ice cold PFA (4%). Histological processing of spinal cords was then conducted as described above.

### High resolution ‘mapping’ microscopy

High resolution images (63x magnification) were captured using a Zeiss Axio Imager M2 microscope equipped with an Apotome.2, which provides an xy-resolution of ~320 nm as tested on fluorescent nano-spheres (data not shown). Illumination was provided by a HXP120 lamp, and images acquired using an MRm camera, with a resultant pixel size of 102.4 nm. PSD95-eGFP-only images were acquired using a 1000 ms exposure time. Three colour images containing PSD95-eGFP and immunohistochemically labelling for GFAP, EAAT2, p-Ezrin, VGLUT2 and VGLUT1 were always captured using exposure times that were kept conserved within datasets produced for comparison. To ‘map’ entire hemi-sections of spinal cords to study anatomical diversity between laminae, single optical sections were captured and tiled across half a transverse section of the spinal cord. Images were stitched together to create whole montage images that were subsequently analysed.

### Super-resolution microscopy

Single channel confocal and gated-stimulated emission depletion (g-STED) microscopy of PSD95-eGFP in the dorsal and ventral horn of the adult spinal cord was performed using the Leica SP5 SMD g-STED microscope available at the Edinburgh Super-Resolution Imaging Consortium hosted by Heriot Watt University. Image acquisition was performed as described previously^4^. Excitation was provided by a CW super-continuum white light laser source at 488 nm to excite eGFP with depletion provided by a 594 nm laser. Images were acquired with a 100×1.4NA STED objective lens with an optical zoom set to provide optimal xy-resolution of ~90 nm^4^. The resultant image pixel size was 19.97 nm. Fluorescence was detected using a Leica Hybrid detector between 500–560 nm, gated at 2–8 ns for g-STED images. Confocal and g-STED images were captured sequentially (confocal then g-STED) from regions of interest.

Multi-colour g-STED microscopy was performed using the Leica SP8 SMD g-STED Microscope, equipped with 592 nm and 775 nm depletion laser lines, with images captured using two HyD detectors. EAAT2 (secondary antibody label of Alexa Fluor 594) and VGLUT1/2 (secondary antibody label of Alexa Fluor 647) were depleted with the 775 nm depletion laser line, and a 0.5–6 ns gating. 775 nm depletion images were acquired before 592 nm depletion images in order to minimise bleaching of the red fluorescence channels with the 592 nm depletion laser. In these data sets, STED images were acquired before Confocal images, in order to obtain the best quality super-resolution images of PSD95 NCs for analysis.

### **Mapping image analysis**

Large scale image analysis of PSD95-eGFP expression along with astrocyte markers and presynaptic markers was performed in ImageJ (FIJI) with customised macros. Spinal cord laminae were delineated manually from images guided by a standard anatomical atlas^99^ and were defined as Rexed’s laminae: I-II, III-IV, V, VI, VII, VIII, IX and X. Whole field intensity was measured from the raw stitched images from each ROI to provide a measure of overall PSD95 expression (a factor of both the number of puncta and the fluorescence intensity of each individual punctum). Puncta were detected using a similar approach to that described by Broadhead *et al*., (2016). Briefly, images were processed sequentially with a background subtraction (10 pixel radius for synaptic puncta EAAT2 and p-Ezrin and 25 pixel radius for GFAP processes) and a Gaussian smoothing filter at 1.5 pixel radius. Automated-thresholding was performed to binarize punctate images (i.e. for synapse markers, EAAT2 or p-Ezrin) using a “Moments” algorithm, as is well suited to punctate analysis^4,100,101^. GFAP images were binarized using “Li” based automated thresholding. The mean fluorescence intensity was recorded by redirecting measurements back to the original raw images. Whether PSD95-eGFP structures were associated with astrocytes or presynaptic terminals was determined by redirecting intensity measurements from binarized PSD95-eGFP masks to binarized images of astrocytic or presynaptic markers. If a binarized EAAT2 or VGLUT structure overlapped with a binarized PSD95 structure by at least 1 pixel (each pixel 102.4 nm) then colocalization was considered true. This stringent method for determining the proximity of astrocytic and synaptic structures helps to ensure that only PSDs with a strong local presence of presynaptic or astrocytic markers are considered synaptic or tripartite synaptic in nature. Additional colocalization analysis was performed in the form of nearest neighbour analysis using the plugin: Distance Analysis (DiAna,^102^). DiAna was used to quantify the association of EAAT2 with synapses by detecting the 10 EAAT2 puncta that were nearest neighbours to each PSD and then calculating the distances between the centre points of PSDs and their neighbouring EAAT2 puncta.

### Super-resolution image analysis

Confocal/g-STED images of PSD95-eGFP alone in Fig. 2 were analysed as described previously^4^^.^ Using Imaris Cell to determine whole PSDs as ‘Cells’ from confocal images, and quantitate PSD95 NCs as ‘nuclei’ within each parent Cell. Analysis of g-STED of PSD95-eGFP along with VGLUT1, VGLUT2 and EAAT2 was performed in ImageJ (FIJI) using processing and quantitative steps analogous to the Imaris Cell technique. Briefly, images were processed using a background subtraction and Gaussian smoothing. They were then binarized for analysis using “Moments” based automated-thresholding, with the exception of VGLUT2 images which required manual adjustment of the threshold in some images to best detect the presynaptic structures. Saved, binarized images of PSD95 PSDs were added to the “ROI Manager” tool within ImageJ, and the following macro analysed each individual PSD95 NC named according to the respective PSD to which it belonged:

*run(“Set Measurements…”, “area mean display redirect*  = *PSD95_Nanocluster image.tif decimal*  = *3”);*

*id*  = *getImageID();*

*setAutoThreshold(“Default”)*;

*for (i*  = *0; i* < *roiManager(“count”);*

*i* + +*) {*

*selectImage(id);*

*roiManager(“select”, i)*;

*run(“Analyze Particles…”, “size*  = *8-Infinity pixel show*  = *Nothing display”);*

*}*

Similar to analysis of colocalisation within our high-resolution microscopy data, VGLUT presynaptic structures and EAAT2 astrocytic structures were deemed to be associated with a PSD if there was overlap of at least 1 pixel (19.97 nm) with the PSD95 PSD structures. The lower resolution confocal images used to visualise PSDs enabled sufficient blurring of PSD95 such that spatially separate structures such as EAAT2 and VGLUT2 captured with g-STED still fall within the binarized realm of their neighbouring diffraction limited PSD. In addition to this colocalization based analysis, DiAna was used to quantify the edge-edge distances of EAAT2 nanodomains to PSDs in g-STED data. FWHM analysis was performed by manually identifying individual substructures from images and calculating the diameters along their apparent minimum and maximum axes from line profiles.

### Data handling and statistical analyses

Data storage was provided by a QNAP TS-879 Pro NAS drive tower with an Intel i3 3.3 GHz Dual core. Data handling and graph preparation was performed in Microsoft Excel 2016. Statistical tests of significance were performed in SPSS Statistics 23 (IBM). Data sets were tested for normality by calculating Shapiro Wilks and Kolmogorov-Smirnov test scores. Factorial analysis of variance (ANOVA) tests with post hoc Tukey’s tests were performed to determine age-dependent and regional differences in synaptic parameters. Separate one-way ANOVAs were performed to ascertain inter-laminae differences within age groups for key synaptic parameters (PSD density, size, intensity). Data sets comparing PSD size with the number of EAAT2 neighbouring puncta, and VGLUT1 and VGLUT2 parameters were analysed with a Two-Sample-T test. Repeated measures ANOVA were run for intensity measurements of PSDs when associated with different components of the tripartite synapse, to account for differences in intensity between mice. Paired T-tests were run for nearest neighbour analysis of EAAT2 and comparisons of its association with PSDs between the true and rotated images. For g-STED analysis of the number of NCs per PSD, Chi squared tests were performed, while size and intensity were analysed using Mann Whitney U non-parametric tests. Data are plotted as mean plus or minus standard error of the mean. Statistical significance is indicated as follows: ns (not significant), *p < *0.05*, **p < *0.01*, ***p < *0.001*.

## Supplementary information

Supplementary Figures.

## Data Availability

The data sets generated and analysed in this study, as well as more in-depth methodological details, are available from the corresponding author upon request.

## References

[1] Tang A-H (2016). A trans-synaptic nanocolumn aligns neurotransmitter release to receptors. Nature.

[2] Newpher TM, Ehlers MD (2008). Glutamate Receptor Dynamics in Dendritic Microdomains. Neuron.

[3] Glickfeld LL, Scanziani M (2006). Distinct timing in the activity of cannabinoid-sensitive and cannabinoid-insensitive basket cells. Nat. Neurosci..

[4] Broadhead MJ (2016). PSD95 nanoclusters are postsynaptic building blocks in hippocampus circuits. Sci. Rep..

[5] MacGillavry HD, Song Y, Raghavachari S, Blanpied TA (2013). Nanoscale Scaffolding Domains within the Postsynaptic Density Concentrate Synaptic AMPA Receptors. Neuron.

[6] Geinisman Y (2000). Structural synaptic modifications associated with hippocampal LTP and behavioral learning. Cereb. Cortex.

[7] Geinisman Y, de Toledo-Morrell L, Morrell F, Persina IS, Rossi M (1992). Age-related loss of axospinous synapses formed by two afferent systems in the rat dentate gyrus as revealed by the unbiased stereological dissector technique. Hippocampus.

[8] Harris KM, Weinberg RJ (2012). Ultrastructure of Synapses in the Mammalian Brain. Cold Spring Harb. Perspect. Biol.

[9] Okabe S, Miwa A, Okado H (2001). Spine formation and correlated assembly of presynaptic and postsynaptic molecules. J. Neurosci..

[10] Collins MO (2006). Molecular characterization and comparison of the components and multiprotein complexes in the postsynaptic proteome. J. Neurochem.

[11] Bayés A, Grant SGN (2009). Neuroproteomics: understanding the molecular organization and complexity of the brain. Nat. Rev. Neurosci..

[12] Bayés À (2012). Comparative study of human and mouse postsynaptic proteomes finds high compositional conservation and abundance differences for key synaptic oroteins. Plos One.

[13] Ryan TJ, Grant SGN (2009). The origin and evolution of synapses. Nat. Rev. Neurosci..

[14] Husi H, Grant SGN (2001). Isolation of 2000-kDa complexes of N-methyl-D-aspartate receptor and postsynaptic density 95 from mouse brain. J. Neurochem..

[15] Fernández, E. *et al*. Targeted tandem affinity purification of PSD-95 recovers core postsynaptic complexes and schizophrenia susceptibility proteins. *Mol. Syst. Biol*. **5** (2009). 10.1038/msb.2009.27 PMC2694677 19455133

[16] Frank RAW (2016). NMDA receptors are selectively partitioned into complexes and supercomplexes during synapse maturation. Nat. Commun..

[17] Frank RA, Grant SG (2017). Supramolecular organization of NMDA receptors and the postsynaptic density. Curr. Opin. Neurobiol..

[18] Nair D (2013). Super-resolution imaging reveals that AMPA receptors inside synapses are dynamically organized in nanodomains regulated by PSD95. J. Neurosci..

[19] Fukata Y (2013). Local palmitoylation cycles define activity-regulated postsynaptic subdomains. J. Cell Biol.

[20] Zhu, F. *et al*. Architecture of the Mouse Brain Synaptome. *Neuron*, 10.1016/j.neuron.2018.07.007 (2018). 10.1016/j.neuron.2018.07.007 PMC6117470 30078578

[21] Harris KM, Landis DM (1986). Membrane structure at synaptic junctions in area CA1 of the rat hippocampus. Neuroscience.

[22] Betley JN (2009). Stringent specificity in the construction of a GABAergic presynaptic inhibitory circuit. Cell.

[23] Witts EC, Zagoraiou L, Miles GB (2014). Anatomy and function of cholinergic C bouton inputs to motor neurons. J. Anat..

[24] Wilson JM, Rempel J, Brownstone RM (2004). Postnatal development of cholinergic synapses on mouse spinal motoneurons. J. Comp. Neurol..

[25] Carlsen EM, Perrier J-F (2014). Purines released from astrocytes inhibit excitatory synaptic transmission in the ventral horn of the spinal cord. Front. Neural Circuits.

[26] Todd AJ (2010). Neuronal circuitry for pain processing in the dorsal horn. Nat. Rev. Neurosci..

[27] Alvarez FJ, Villalba RM, Zerda R, Schneider SP (2004). Vesicular glutamate transporters in the spinal cord, with special reference to sensory primary afferent synapses. J. Comp. Neurol..

[28] Brumovsky P, Watanabe M, Hökfelt T (2007). Expression of the vesicular glutamate transporters-1 and -2 in adult mouse dorsal root ganglia and spinal cord and their regulation by nerve injury. Neuroscience.

[29] Larsson, M. & Broman, J. Synaptic organization of VGLUT3 expressing low-threshold mechanosensitive C fiber terminals in the rodent spinal cord. *eNeuro* **6** (2019). 10.1523/ENEURO.0007-19.2019 PMC6378328 30783617

[30] Khakh BS, Sofroniew MV (2015). Diversity of astrocyte functions and phenotypes in neural circuits. Nat. Neurosci..

[31] Clarke LE, Barres BA (2013). Emerging roles of astrocytes in neural circuit development. Nat. Rev. Neurosci..

[32] Derouiche A, Anlauf E, Aumann G, Mühlstädt B, Lavialle M (2002). Anatomical aspects of glia–synapse interaction: the perisynaptic glial sheath consists of a specialized astrocyte compartment. J. Physiol..

[33] Perea G, Navarrete M, Araque A (2009). Tripartite synapses: astrocytes process and control synaptic information. Trends Neurosci..

[34] Covelo A, Araque A (2018). Neuronal activity determines distinct gliotransmitter release from a single astrocyte. Elife.

[35] Ventura R, Harris KM (1999). Three-Dimensional Relationships between Hippocampal Synapses and Astrocytes. J. Neurosci..

[36] Haber M, Zhou L, Murai KK (2006). Cooperative Astrocyte and Dendritic Spine Dynamics at Hippocampal Excitatory Synapses. J. Neurosci..

[37] Acton D, Miles GB (2015). Stimulation of Glia Reveals Modulation of Mammalian Spinal Motor Networks by Adenosine. Plos One.

[38] Witts EC, Nascimento F, Miles GB (2015). Adenosine-mediated modulation of ventral horn interneurons and spinal motoneurons in neonatal mice. J. Neurophysiol..

[39] Kopach O (2011). Inflammation alters trafficking of extrasynaptic AMPA receptors in tonically firing lamina II neurons of the rat spinal dorsal horn. Pain.

[40] Ikeda H, Kiritoshi T, Murase K (2012). Contribution of microglia and astrocytes to the central sensitization, inflammatory and neuropathic pain in the juvenile rat. Mol. Pain.

[41] Vigneault, É. *et al*. Distribution of vesicular glutamate transporters in the human brain. *Front. Neuroanat*. **9** (2015). 10.3389/fnana.2015.00023 PMC4350397 25798091

[42] Hegarty DM, Tonsfeldt K, Hermes SM, Helfand H, Aicher SA (2010). Differential localization of vesicular glutamate transporters and peptides in corneal afferents to trigeminal nucleus caudalis. J. Comp. Neurol..

[43] Todd AJ (2003). The expression of vesicular glutamate transporters VGLUT1 and VGLUT2 in neurochemically defined axonal populations in the rat spinal cord with emphasis on the dorsal horn. Eur. J. Neurosci..

[44] Ni Y (2014). Characterization of Long Descending Premotor Propriospinal Neurons in the Spinal Cord. J. Neurosci..

[45] Lagerström MC (2010). VGLUT2-dependent sensory neurons in the TRPV1 population regulate pain and itch. Neuron.

[46] Du Beau A (2012). Neurotransmitter phenotypes of descending systems in the rat lumbar spinal cord. Neuroscience.

[47] Acton, D., Broadhead, M. J. & Miles, G. B. Modulation of spinal motor networks by astrocyte-derived adenosine is dependent on D1-like dopamine receptor signalling. *J. Neurophysiol*., 10.1152/jn.00783.2017 (2018). 10.1152/jn.00783.2017 PMC6171060 29790837

[48] DeSilva TM, Borenstein NS, Volpe JJ, Kinney HC, Rosenberg PA (2012). Expression of EAAT2 in neurons and protoplasmic astrocytes during human cortical development. J. Comp. Neurol..

[49] Lavialle M (2011). Structural plasticity of perisynaptic astrocyte processes involves ezrin and metabotropic glutamate receptors. Proc. Natl. Acad. Sci..

[50] Panatier A, Arizono M, Nagerl UV (2014). Dissecting tripartite synapses with STED microscopy. Philos. Trans. R. Soc. B Biol. Sci.

[51] Heller JP, Rusakov DA (2017). The Nanoworld of the Tripartite Synapse: Insights from Super-Resolution Microscopy. Front. Cell. Neurosci.

[52] Dosemeci A (2007). Composition of the Synaptic PSD-95 Complex. Mol. &amp; Cell. Proteomics.

[53] Bayés A (2011). Characterization of the proteome, diseases and evolution of the human postsynaptic density. Nat. Neurosci..

[54] Schitine C, Nogaroli L, Costa MR, Hedin-Pereira C (2015). Astrocyte heterogeneity in the brain: from development to disease. Front. Cell. Neurosci.

[55] Chaboub LS, Deneen B (2012). Developmental Origins of Astrocyte Heterogeneity: The Final Frontier of CNS Development. Dev Neurosci.

[56] Emsley JG, Macklis JD (2006). Astroglial heterogeneity closely reflects the neuronal-defined anatomy of the adult murine CNS. Neuron Glia Biol.

[57] Dvorzhak A, Helassa N, Török K, Schmitz D, Grantyn R (2019). Single Synapse Indicators of Impaired Glutamate Clearance Derived from Fast iGlu u Imaging of Cortical Afferents in the Striatum of Normal and Huntington (Q175) Mice. J. Neurosci..

[58] Foster JB (2018). Pyridazine-derivatives Enhance Structural and Functional Plasticity of Tripartite Synapse Via Activation of Local Translation in Astrocytic Processes. Neuroscience.

[59] Yamada K (1998). Glutamate transporter GLT-1 is transiently localized on growing axons of the mouse spinal cord before establishing astrocytic expression. J. Neurosci..

[60] Weng DY (2008). Promiscuous recombination of LoxP alleles during gametogenesis in cornea Cre driver mice. Mol. Vis..

[61] Spacek J (1985). Three-dimensional analysis of dendritic spines. III. Glial sheath. Anat. Embryol. (Berl).

[62] Tarabal O (2014). Mechanisms involved in spinal cord central synapse loss in a mouse model of spinal muscular atrophy. J. Neuropathol. Exp. Neurol..

[63] Chen L, Hamaguchi K, Hamada S, Okado N (1997). Regional differences of serotonin-mediated synaptic plasticity in the chicken spinal cord with development and aging. J. Neural Transplant. Plast..

[64] Chiu CS, Kartalov E, Unger M, Quake S, Lester HA (2001). Single-molecule measurements calibrate green fluorescent protein surface densities on transparent beads for use with ‘knock-in’ animals and other expression systems. J. Neurosci. Methods.

[65] Tao Y-X, Huang Y-Z, Mei L, Johns R (2000). Expression of PSD-95/SAP90 is critical for N-methyl-d-aspartate receptor-mediated thermal hyperalgesia in the spinal cord. Neuroscience.

[66] Tao F, Su Q, Johns RA (2008). Cell-permeable Peptide Tat-PSD-95 PDZ2 Inhibits Chronic Inflammatory Pain Behaviors in Mice. Mol. Ther..

[67] Tao F (2001). Knockdown of PSD-95/SAP90 delays the development of neuropathic pain in rats. Neuroreport.

[68] Arbuckle MI (2010). The SH3 domain of postsynaptic density 95 mediates inflammatory pain through phosphatidylinositol-3-kinase recruitment. EMBO Rep..

[69] Garry EM (2003). Neuropathic Sensitization of Behavioral Reflexes and Spinal NMDA Receptor/CaM Kinase II Interactions Are Disrupted in PSD-95 Mutant Mice. Curr. Biol..

[70] Abbinanti MD, Zhong G, Harris-Warrick RM (2012). Postnatal emergence of serotonin-induced plateau potentials in commissural interneurons of the mouse spinal cord. J. Neurophysiol..

[71] Clarac F, Vinay L, Cazalets JR, Fady JC, Jamon M (1998). Role of gravity in the development of posture and locomotion in the neonatal rat. Brain Res. Brain Res. Rev..

[72] Liu KKL, Hagan MF, Lisman JE (2017). Gradation (approx. 10 size states) of synaptic strength by quantal addition of structural modules. Philos. Trans. R. Soc. Lond. B. Biol. Sci.

[73] Hruska M, Henderson N, Le Marchand SJ, Jafri H, Dalva MB (2018). Synaptic nanomodules underlie the organization and plasticity of spine synapses. Nat. Neurosci..

[74] Specht CG (2013). Quantitative Nanoscopy of Inhibitory Synapses: Counting Gephyrin Molecules and Receptor Binding Sites. Neuron.

[75] Crosby KC (2019). Nanoscale Subsynaptic Domains Underlie the Organization of the Inhibitory Synapse. Cell Rep..

[76] Brännström T (1993). Quantitative synaptology of functionally different types of cat medial gastrocnemius α‐motoneurons. J. Comp. Neurol..

[77] Persson S, Havton LA (2008). Differential synaptic inputs to the cell body and proximal dendrites of preganglionic parasympathetic neurons in the rat conus medullaris. Neuroscience.

[78] Walmsley B, Alvarez FJ, Fyffe REW (1998). Diversity of structure and function at mammalian central synapses. Trends Neurosci..

[79] Caroni P, Donato F, Muller D (2012). Structural plasticity upon learning: regulation and functions. Nat. Rev. Neurosci..

[80] Brumovsky PR (2013). VGLUTs in Peripheral Neurons and the Spinal Cord: Time for a Review. ISRN Neurol.

[81] Rèthelyi M, Light AR, Perl ER (1989). Synaptic ultrastructure of functionally and morphologically characterized neurons of the superficial spinal dorsal horn of cat. J. Neurosci..

[82] Ribeiro-da-Silva A, Coimbra A (1982). Two types of synaptic glomeruli and their distribution in laminae I-III of the rat spinal cord. J. Comp. Neurol..

[83] Wojcik SM (2004). An essential role for vesicular glutamate transporter 1 (VGLUT1) in postnatal development and control of quantal size. Proc. Natl. Acad. Sci..

[84] Fremeau RT (2001). The expression of vesicular glutamate transporters defines two classes of excitatory synapse. Neuron.

[85] Fremeau RT (2004). Vesicular glutamate transporters 1 and 2 target to functionally distinct synaptic release sites. Science (80-).

[86] Varoqui H, Schäfer MKH, Zhu H, Weihe E, Erickson JD (2002). Identification of the differentiation-associated Na+/PI transporter as a novel vesicular glutamate transporter expressed in a distinct set of glutamatergic synapses. J. Neurosci..

[87] Weston MC, Nehring RB, Wojcik SM, Rosenmund C (2011). Interplay between VGLUT isoforms and endophilin A1 regulates neurotransmitter release and short-term plasticity. Neuron.

[88] Masch J-M (2018). Robust nanoscopy of a synaptic protein in living mice by organic-fluorophore labeling. Proc. Natl. Acad. Sci. USA..

[89] Choquet D, Triller A (2013). The dynamic synapse. Neuron.

[90] Wegner W, Mott AC, Grant SGN, Steffens H, Willig KI (2018). *In vivo* STED microscopy visualizes PSD95 sub-structures and morphological changes over several hours in the mouse visual cortex. Sci. Rep..

[91] Junge CE (2004). Protease-activated receptor-1 in human brain: localization and functional expression in astrocytes. Exp. Neurol..

[92] Beamer E, Kovács G, Sperlágh B (2017). ATP released from astrocytes modulates action potential threshold and spontaneous excitatory postsynaptic currents in the neonatal rat prefrontal cortex. Brain Res. Bull..

[93] Sweeney, A. M. *et al*. PAR1 activation induces rapid changes in glutamate uptake and astrocyte morphology. *Sci. Rep*. **7** (2017). 10.1038/srep43606 PMC5335386 28256580

[94] Sun Q, Turrigiano GG (2011). PSD-95 and PSD-93 play critical but distinct roles in synaptic scaling up and down. J. Neurosci..

[95] Ota Y, Zanetti AT, Hallock RM (2013). The role of astrocytes in the regulation of synaptic plasticity and memory formation. Neural Plast..

[96] Herron LR, Miles GB (2012). Gender-specific perturbations in modulatory inputs to motoneurons in a mouse model of amyotrophic lateral sclerosis. Neuroscience.

[97] Sloan SA, Barres BA (2014). Mechanisms of astrocyte development and their contributions to neurodevelopmental disorders. Curr. Opin. Neurobiol..

[98] Sunico CR (2011). Reduction in the Motoneuron Inhibitory/Excitatory Synaptic Ratio in an Early-Symptomatic Mouse Model of Amyotrophic Lateral Sclerosis. Brain Pathol..

[99] Watson, C., Paxinos, G., Kayalioglu, G. & Christopher & Dana Reeve Foundation. *The spinal cord: a Christopher and Dana Reeve Foundation text and atlas*. (Elsevier/Academic Press, 2009).

[100] Glebov, O. O., Cox, S., Humphreys, L. & Burrone, J. Neuronal activity controls transsynaptic geometry. *Sci. Rep*. **6** (2016). 10.1038/srep22703 PMC4782104 26951792

[101] Castells-Nobau, A. *et al*. Two algorithms for high-throughput and multi-parametric quantification of drosophila neuromuscular junction morphology. *J. Vis. Exp*. **2017** (2017). 10.3791/55395 PMC5607876 28518121

[102] Gilles J-F, Dos Santos M, Boudier T, Bolte S, Heck N (2017). DiAna, an ImageJ tool for object-based 3D co-localization and distance analysis. Methods.

