# Author Correction: Nanostructural Diversity of Synapses in the Mammalian Spinal Cord

**DOI:** 10.1038/s41598-021-02575-7

**Published:** 2021-11-24

**Authors:** Matthew J. Broadhead, Calum Bonthron, Lauren Arcinas, Sumi Bez, Fei Zhu, Frances Goff, Jonathan Nylk, Kishan Dholakia, Frank Gunn-Moore, Seth G. N. Grant, Gareth B. Miles

**Affiliations:** 1 School of Psychology and Neuroscience, University of St Andrews, St Andrews, UK; 2 Edinburgh Super-Resolution Imaging Consortium, Heriot Watt University, Edinburgh, UK; 3 Genes to Cognition Programme, Centre for Clinical Brain Sciences, University of Edinburgh, Edinburgh, UK; 4 School of Biology, University of St Andrews, St Andrews, UK; 5 SUPA, School of Physics and Astronomy, University of St Andrews, St Andrews, UK; 6 Centre for Biophotonics, North Haugh, University of St Andrews, St Andrews, UK

Correction to: *Scientific Reports* https://doi.org/10.1038/s41598-020-64874-9, published online 18 May 2020

The original version of this Article contained an error in Figure 1D, where the y-axis label “Puncta Density (Number per 200 μm^2^)” was incorrectly given as “Puncta Density (Number per 20 μm^2^)”.

The original Figure 1 and accompanying legend appear below.

**Figure 1:**
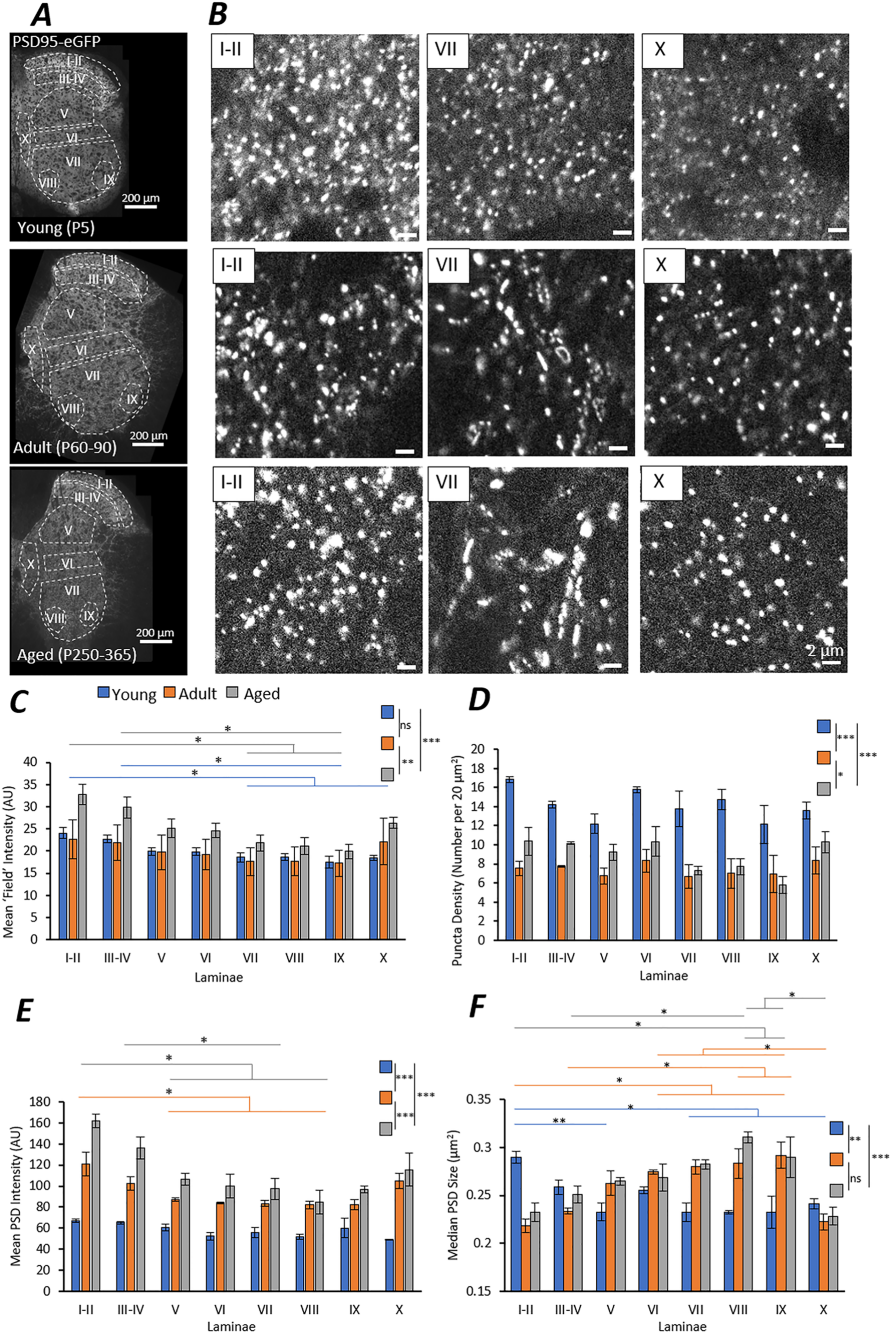
Mapping PSD95-eGFP PSDs in the Mouse Spinal Cord. (**A**) High magnification tiled imaging was performed on half-spinal cord sections from PSD95-eGFP mice from three age groups (n = 3 mice per age group). Quantitative analysis was performed at the single synapse level to study synaptic diversity between laminae and across age groups. (**B**) Example images of PSD95-eGFP PSDs in three laminae from each age group. Note the formation of large elongated PSDs in VII of Adult and Aged mice. (**C**) The mean ‘field’ intensity of PSD95-eGFP labelling was quantified from entire delineated laminae at each age. Age groups are colour coded as Young (blue), Adult (orange) and Aged (grey). Colour-coded lines indicate statistical significance between laminae within an age group following a post-hoc test. The PSD density (number per unit area) (**D**), the mean fluorescence intensity of PSDs (**E**) and the median PSD size (**F**) is also plotted for each lamina in each age group.

The original Article has been corrected.

