# Supplementary material for: Nanostructural Diversity of Synapses in the Mammalian Spinal Cord
Source: Sci Rep. 2020 May 18;10:8189. doi: 10.1038/s41598-020-64874-9 (PMC7235094; doi:10.1038/s41598-020-64874-9)
Supplement: Supplementary file 1 — Supplementary Figures. [file 41598_2020_64874_MOESM1_ESM.pdf]

Title: Nanostructural Diversity of Synapses in the Mammalian Spinal Cord.

Authors: Matthew J. Broadhead[*1,2]; Calum Bonthron[*1]; Lauren Arcinas[1]; Sumi Bez[1]; Fei Zhu[3]; Frances Goff[4]; Jonathan Nylk[5]; Kishan Dholakia[5]; Frank Gunn-Moore[4]; Seth G.N. Grant[3]; Gareth B. Miles[†1].

* Co-First Author

1. School of Psychology and Neuroscience, University of St Andrews, St Andrews, UK.
2. Edinburgh Super-Resolution Imaging Consortium, Heriot Watt University, Edinburgh, UK.
3. Genes to Cognition Programme, Centre for Clinical Brain Sciences, University of Edinburgh, Edinburgh, UK,
4. School of Biology, University of St Andrews, St Andrews, UK.
5. SUPA, School of Physics and Astronomy, University of St Andrews, St Andrews, UK.

† Email: gbm4@st-andrews.ac.uk

Supplementary Figure 1

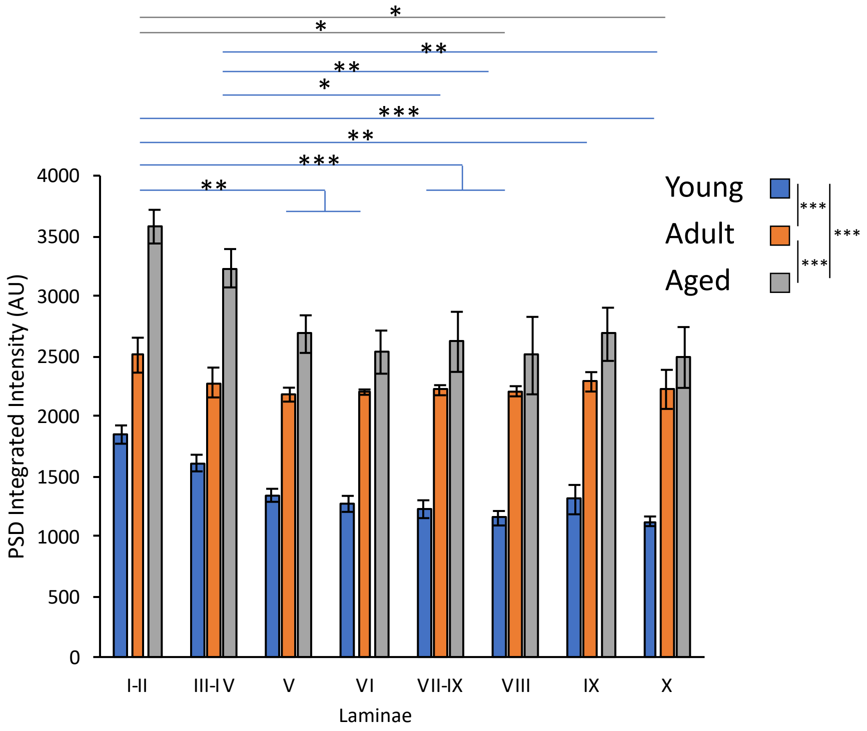

SI. Fig. 1. Additional Analysis of PSD95-eGFP Expression in the Mouse Spinal Cord.
Bar chart depicting the integrated intensity of PSD95-eGFP PSDs in each spinal laminae for each age group.

Supplementary Figure 2

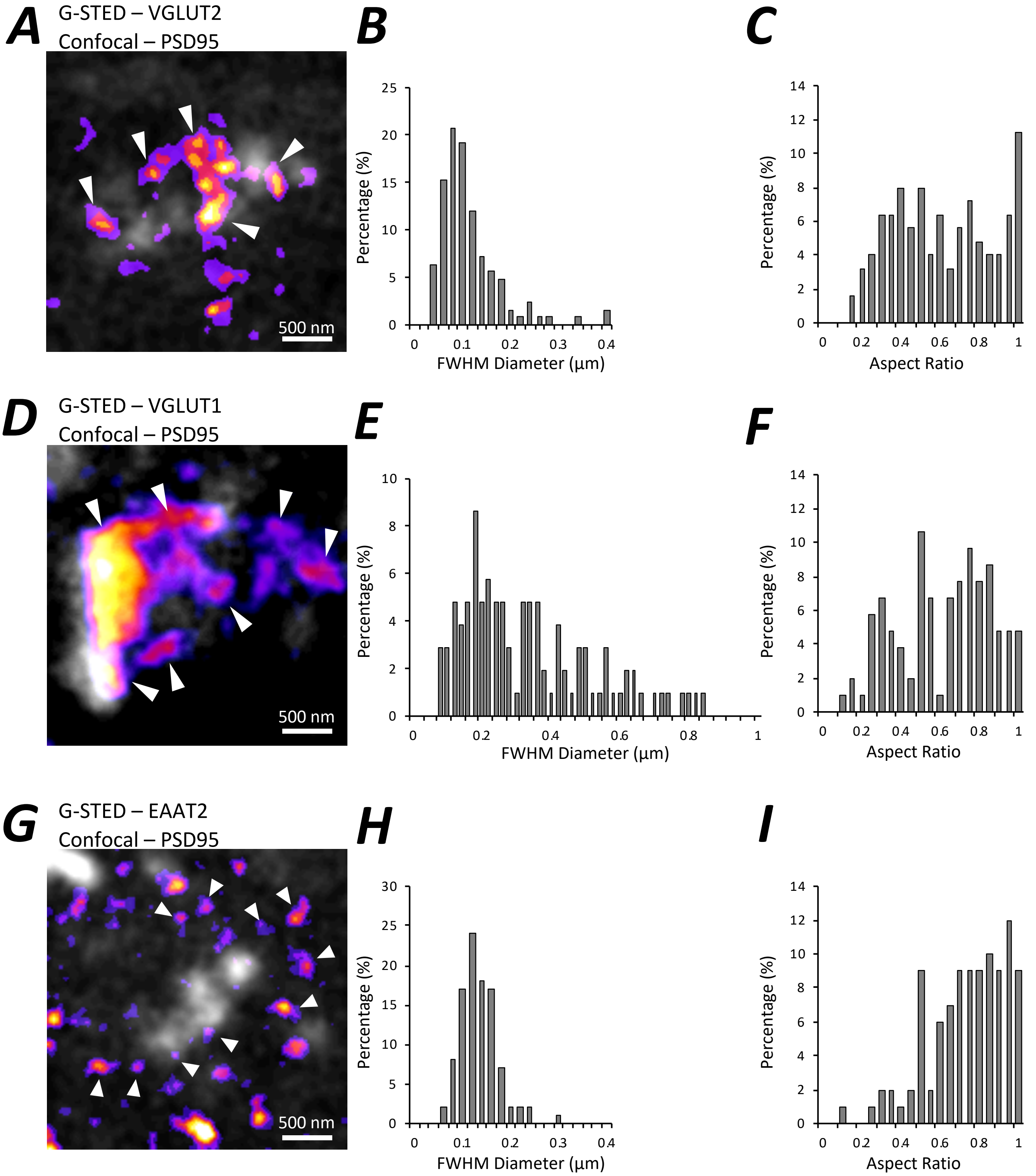

SI. Fig. 2. SR-Microscopy of VGLUT2, VGLUT1 and EAAT2.
A. Image of PSD95 PSD (grey-scale, confocal) and VGLUT2 (fire-scale, g-STED). Arrows highlight examples of sub-structures in a VGLUT2 bouton. **B**. Histogram of the diameters of VGLUT2 substructures. **C**. Histogram of the aspect ratio (shape factor) of VGLUT2 substructures. **D**. Image of PSD95 PSD (grey-scale, confocal) and VGLUT1 (fire-scale, g-STED). Arrows highlight examples of sub-structures in a VGLUT1 bouton. **E.** Histogram of the diameters of VGLUT1 substructures. **F**. Histogram of the aspect ratio (shape factor) of VGLUT1 substructures.
G. Image of PSD95 PSD (grey-scale, confocal) and EAAT2 (fire-scale, g-STED). Arrows highlight examples of EAAT2 nanodomains. **H**. Histogram of the diameters of EAAT2 substructures. **I**. Histogram of the aspect ratio (shape factor) of EAAT2 substructures.

Supplementary Figure 3

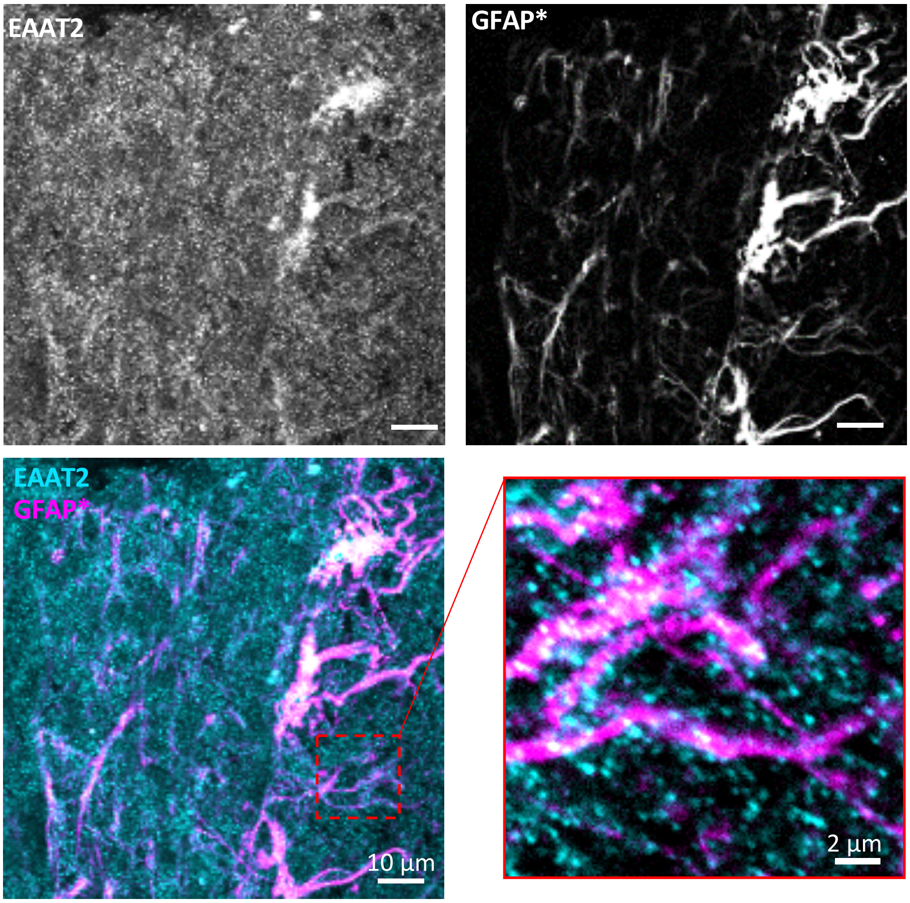

SI. Fig. 3. GFAP and EAAT2 Labelling for Astrocytes

Images were captured from the ventral horn of the adult mouse spinal cord using two astrocytic markers, EAAT2 and GFAP. EAAT2 is strongly expressed in GFAP-positive cell bodies and processes. There are also a large number of EAAT2 puncta that do not colocalise with GFAP, and may therefore label the finer extremities of the astrocyte which are more likely to contact synapses. *GFAP labelling depicted here was performed using an antibody raised in chicken, whilst other GFAP labelling was obtained using an antibody raised in rabbit.

Supplementary Figure 4

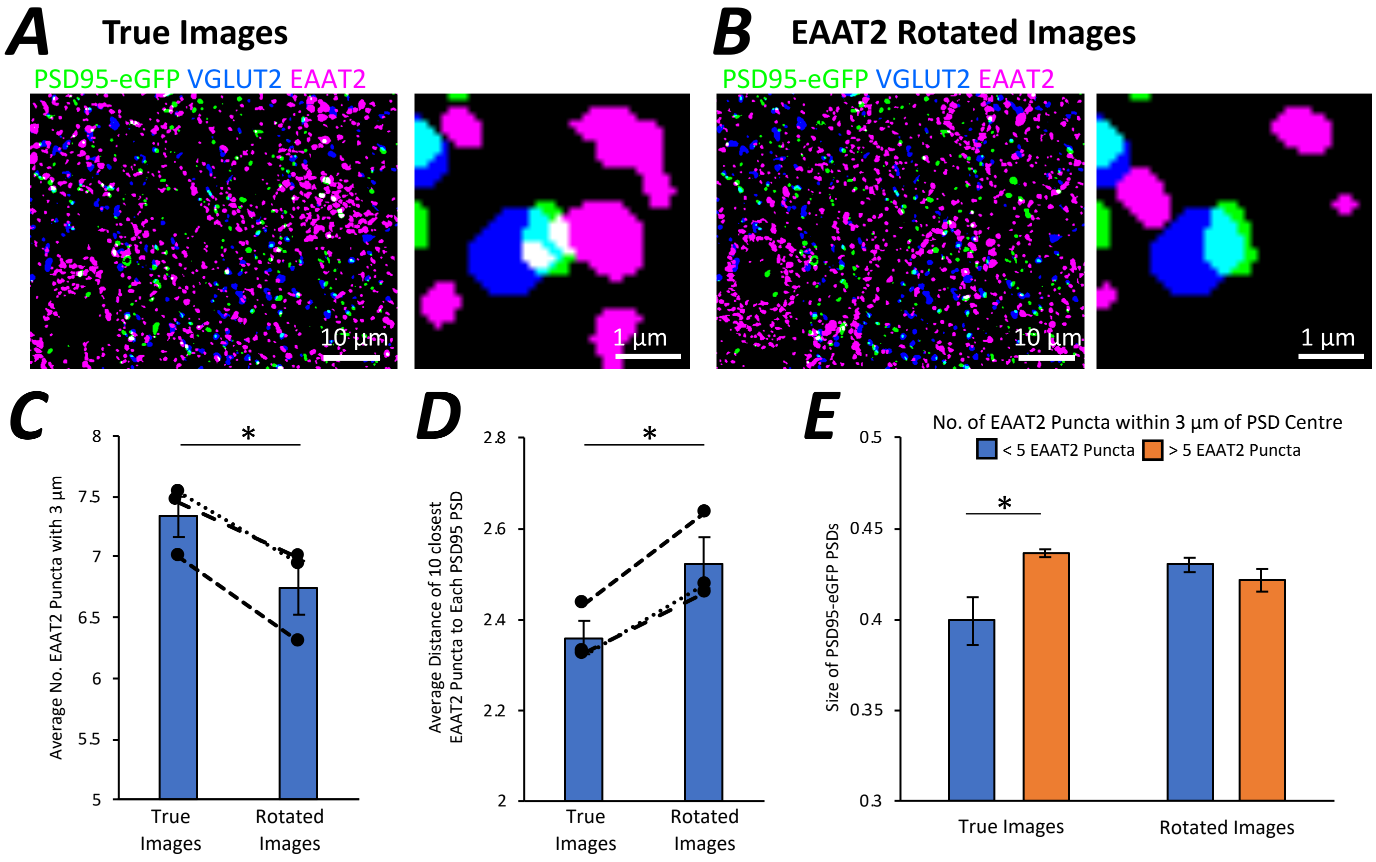

SI. Fig. 4. Nearest Neighbour Analysis of Images of Tripartite Synapses and Rotated Control Images.

A. Processed and binarized images of PSD95-eGFP, VGLUT2 and EAAT2 are displayed from the original image (“True Images”), with a zoomed in panel showing an individual synapse. B. The same image as in panel A is used, but with EAAT2 images rotated 180° to produce “Rotated” images as a control to assess random association between components of tripartite synapses. C. Bar chart displaying the average number of EAAT2 particles within a 3 µm radius of the centre of each PSD95-eGFP PSD in the True and Rotated images. Dashed lines show the results for each mouse, pairing the results from the True images to the Rotated images. D. Bar chart displaying the average distance of the nearest 10 EAAT2 particles to the centre of each PSD95-eGFP PSD in the True and Rotated images. Dashed lines show the results for each mouse, pairing the results from the True images to the Rotated images. E. Bar chart showing the size of PSD95-eGFP PSDs increases when associated with more than 5 EAAT2 puncta, an effect which is not observed in the Rotated control images.
